# CCNY-mediated phosphorylation and TET2-BACH1-driven DNA demethylation activate PRC1 to augment NSCLC progression

**DOI:** 10.1186/s13046-025-03472-x

**Published:** 2025-07-15

**Authors:** Dayu Huang, Xianglin Chu, Chunxiao Wu, Xuan Wang, Mengkun Shi, Xiaofeng Chen, Yubao Lyu, Dapeng Li, Xuyu Gu

**Affiliations:** 1 Department of Thoracic Surgery, Huashan Hospital of Fudan University, Shanghai, 200040 P.R. China; 2 Department of Thoracic Surgery, LongHua Hospital of Shanghai University of Traditional Chinese Medicine, Shanghai, 200126 P.R. China; 3 Department of Integrative Medicine, Huashan Hospital of Fudan University, Shanghai, 200040 P.R. China; 4 Department of Oncology, The First Affiliated Hospital of Soochow University, Suzhou, 215006 Jiangsu P.R. China; 5 Department of Oncology, Shanghai Pulmonary Hospital, School of Medicine, Tongji University, Shanghai, 200433 China

**Keywords:** NSCLC, PRC1, CCNY, TET2, BACH1, DNA demethylation

## Abstract

**Background:**

The protein regulator of cytokinesis 1 (PRC1) is a prognostic marker characterized by low DNA methylation in lung cancer. This study aims to examine the function of PRC1 in non-small cell lung cancer (NSCLC) cells and investigates its regulatory mechanisms.

**Methods:**

PRC1 expression in NSCLC cells was assessed using qPCR and western blot analyses. Loss- and gain-of-function assays of PRC1 were performed in NSCLC cells to analyze its effect on cell cycle progression and growth. Genetic knockdown or pharmaceutical inhibition of cyclin Y (CCNY), tet methylcytosine dioxygenase 2 (TET2), and BTB domain and CNC homolog 1 (BACH1) was conducted to analyze their influence on PRC1 phosphorylation or transcription. Subcutaneous xenograft and orthotopic isograft tumor models were generated for in vivo verification. Tissue microarray (TMA) and bioinformatics analyses were employed to evaluate the clinical prognostic value of CCNY, TET2, and PRC1 in NSCLC.

**Results:**

PRC1 was highly expressed in NSCLC cells. Silencing either PRC1 or CCNY, which promotes PRC1 phosphorylation, substantially reduced cell growth in vitro, impaired spindle formation, promoted G2/M phase cell cycle arrest, increased multi-nucleated cells, and weakened tumorigenic activity of cells. Moreover, TET2 was found to induce DNA demethylation of PRC1 and activate its transcription by interacting with BACH1. Inhibition of TET2, BACH1, or the PLK1-PRC1 interaction weakened the tumorigenic potential of NSCLC cells in vivo. The TMA analysis revealed increased levels of CCNY, TET2, and phosphorylated PRC1 in tumor tissues. Bioinformatics analyses suggested that these molecules were correlated with unfavorable prognosis in NSCLC patients.

**Conclusion:**

This study demonstrates a critical oncogenic role of PRC1 in NSCLC. CCNY, which modulates PRC1 phosphorylation, and the TET2-BACH1 cascade, which modulates demethylation and transcription of PRC1, may serve as promising targets for NSCLC management.

**Supplementary Information:**

The online version contains supplementary material available at 10.1186/s13046-025-03472-x.

## Introduction

According to the 2025 cancer statistics, lung cancer represents the second most common cancer but the leading cause of cancer fatalities in the USA, which causes nearly 2.5 times more deaths than second-ranking colorectal cancer [1]. Non-small cell lung cancer (NSCLC) constitutes about 85% of all lung cancer diagnoses [2]. Unfortunately, only 20–25% of patients are diagnosed at an early stage, when curative surgical intervention is a viable option [3]. Most patients are diagnosed at advanced stages, missing the opportunity for potentially curative surgery [4]. In these cases, alternative treatment strategies such as chemotherapy, radiotherapy, immunotherapy, and molecularly targeted therapies, either as monotherapies or in combination, are necessary, though their effectiveness is often limited [5]. Alarmingly, even those diagnosed at earlier stages and treated optimally may still face recurrence and mortality due to the disease [6]. Consequently, there is a growing need for the development of novel therapeutic agents targeting additional pathogenic genes.

Our prior research identified the protein regulator of cytokinesis 1 (PRC1) as a significant risk factor in lung adenocarcinoma (LUAD), the most common subtype of NSCLC [7]. First described by Jiang et al. in 1998, PRC1 is a substrate for cyclin-dependent kinases (CDKs) associated with the mitotic spindle [8]. It fulfills critical functions in regulating the polarization of parallel microtubules and the assembly of the contractile ring, thereby contributing to spindle stability, chromosome segregation, and the completion of cytokinesis [9, 10]. During interphase, PRC1 is primarily located in the nucleus. However, as mitosis progresses, it associates with spindle microtubules and relocates to the central region of the spindle [8, 11]. Given its essential role in cytokinesis, emerging studies have increasingly highlighted PRC1’s involvement in tumorigenesis [12, 13], including in the context of NSCLC, where it has been proposed as a promising therapeutic target [14, 15].

In a prior study, Hernández-Ortega et al. uncovered an active CDK16/cyclin Y (CCNY) complex that regulates the phosphorylation and activity of PRC1. Their work showed that inhibiting CDK16 leads to dephosphorylation of PRC1 and its re-localization to the nucleus during interphase [16]. CCNY, the key cyclin binding partner responsible for activating CDK16 [17, 18], has been shown to influence cell proliferation and tumor progression in NSCLC [19]. This finding raised our interest in investigating whether CCNY plays a role in regulating PRC1 phosphorylation and its involvement in NSCLC progression.

We have previously [7] reported abnormal DNA methylation patterns in LUAD. DNA methylation and hydroxymethylation (also known as demethylation) are among the most studied and significant epigenetic modifications, playing critical roles in cellular development, biology, and cancer progression [20, 21]. DNA methylation involves attaching a methyl group to the 5ʹ-position of cytosine residues, typically within CpG dinucleotides, whereas DNA hydroxymethylation involves the addition of a hydroxymethyl group, resulting in 5-hydroxymethylcytosine (5hmC) [22]. Promoter methylation usually leads to gene silencing, either by preventing transcription factor binding or by recruiting repressive chromatin remodeling complexes, while hydroxymethylation generally promotes gene activation [22]. The enzyme tet methylcytosine dioxygenase 2 (TET2), one of the three TET family members (TET1, TET2, and TET3), plays a pivotal role in the oxidation of 5-methylcytosine (5mC) to 5hmC [23]. TET2 has been extensively investigated for its role in epigenetics and its association with various cancers [24]. This study aims to explore how TET2 regulates PRC1 demethylation and its impact on the progression of NSCLC.

## Materials and methods

### Cells and treatment

Human NSCLC cell lines H1299, H1975, A549, and Calu-3, and normal human lung epithelial cells BEAS-2B, were provided by SUNNCELL Biotechnologies (Wuhan, Hubei, China). Cells were maintained in complete RPMI-1640 (Gibco, Thermo Fisher Scientific, Rockford, IL, USA) supplemented with 10% fetal bovine serum (FBS) and 1% antibiotics. The cells were kept in a 37℃, 5% CO_2_ incubator.

For drug treatment experiments, cells were cultured until they reached the logarithmic growth phase, then transferred to serum-free medium with appropriate drug concentrations. Drugs included the DNA methylation antagonist 5-Azacytidine (5-Aza; Sigma-Aldrich, Merck KGaA, Darmstadt, Germany), TET antagonist Bobcat339 (MedChemExpress [MCE], Monmouth Junction, NJ, USA), BTB domain and CNC homolog 1 (BACH1) antagonist HPPE (MCE), and PLK1-PRC1 antagonist PLK1-IN-10 (MCE). After treatment for 24–72 h, the cells were harvested.

Gene knockdown was performed using short hairpin (sh) RNA of PRC1 (PRC1-kd), CCNY (CCNY-kd), or TET2 (TET2-kd). the shRNA was cloned into the pLKO.1 vector and co-transfected with packaging plasmids (psPAX2 and pMD2.G, #12260 and #12259, Addgene, Cambridge, MA, USA) into 293T cells for lentivirus production. The viral supernatant was collected, filtered, and used to infect target cells. Puromycin (2 µg/mL) was employed to select positive clones for 3–7 d. Gene overexpression was achieved by cloning the full-length cDNA of the target genes (PRC1, CCNY, or BACH1) into the pLenti-CMV-Puro vectors (Addgene#17448). The lentiviral vectors were prepared and used to infect NSCLC cells. After selection using puromycin, the positive clones were expanded and verified by quantitative polymerase chain reaction (qPCR) or western blot (WB) analysis.

### Construction and functional validation of PRC1 phospho-deficient mutants

To identify functionally relevant phosphorylation sites on PRC1, NetPhos 3.1 was used to predict serine/threonine residues with high phosphorylation potential. Among the top-ranked sites, T429, S431, and T495 were selected for functional validation. Site-directed mutagenesis was performed using the QuikChange Lightning Site-Directed Mutagenesis Kit (#210518, Agilent Technologies, Palo Alto, CA, USA) based on a full-length PRC1 cDNA cloned into the pLenti-CMV-Puro vector (Addgene plasmid #17448). Mutant constructs (T429A, S431A, T495A) were confirmed by Sanger sequencing. Lentiviruses were produced by co-transfecting 293T cells with each PRC1 variant construct and packaging plasmids psPAX2 and pMD2.G (Addgene #12260 and #12259) using Lipofectamine 3000 (#L3000008, Invitrogen, Thermo Fisher Scientific). Supernatants were collected at 48 and 72 h, filtered through a 0.45 μm filter, and used to infect PRC1-knockdown or PRC1-knockout H1299 cells, followed by puromycin selection (2 µg/mL) for 5–7 d.

For rescue assays, PRC1-knockout (PRC1^ko^) H1299 cells were generated by CRISPR-Cas9 targeting (guide sequence: 5ʹ-GCAGGATGTGAAGACACGCG-3ʹ), and stable clones were confirmed by genomic PCR and WB analysis. Cells were transfected with PRC1-WT or variant constructs with or without CCNY overexpression plasmid (pcDNA3.1-CCNY-HA) using Lipofectamine 3000. After 48–72 h, protein lysates were collected for WB of phospho-PRC1 (custom antibody or phospho-serine), and colony formation was assessed over 14 d by seeding 500 cells/well in six-well plates.

### RNA isolation and quantification

Total RNA was isolated utilizing the TRI reagent (Invitrogen), followed by RNA concentration and purity assessment utilizing a NanoDrop (Thermo Fisher Scientific). The HiScript III RT SuperMix (Vazyme Biotech Co., Ltd, Nanjing, Jiangsu, China) was employed for cDNA synthesis. The PCR reaction mixture (20 µL) included 10 µL ChamQ Universal SYBR qPCR Master Mix (Vazyme), 1 µL each of forward and reverse primers (10 µM), 2 µL cDNA, and 7 µL nuclease-free water. qPCR was performed using the QuantStudio 6 system (Applied Biosystems, Inc., Carlsbad, CA, USA) Relative expression levels, with GAPDH applied as the control gene, were evaluated using the 2^−ΔΔCt^ method.

### Western blot (WB) analysis

The treated cells were lysed on ice utilizing RIPA buffer and centrifuged, and the supernatant was quantified using a BCA assay (Thermo Fisher Scientific). Subsequently, 30 µg of protein was mixed with 5× SDS sample buffer and denatured at 95℃ for 10 min. Denatured Proteins were separated by 10% gel electrophoresis and loaded to a PVDF membrane. After blocking with 5% bovine serum albumin (BSA) for 1 h, the membrane was probed with antibodies (PRC1, phospho-PRC1, TET2, CCNY, 1:1000 dilution, Abcam Inc., Cambridge, MA, USA) overnight. The next day, HRP-conjugated IgG (1:5000, Cell Signaling Technology [CST], Beverly, MA, USA) were applied for 1 h. Signal bands were developed utilizing ECL reagent and the signal intensity was assessed using Image J.

### Colony formation assay

Exponentially growing cells were resuspended. Subsequently, 500 cells were seeded in each well of a six-well plate with 2 mL medium per well. After 14 d, the medium was discarded, and cells were washed, fixed with 4% paraformaldehyde, and subjected to 0.1% crystal violet (Solarbio Science & Technology Co., Ltd., Beijing, China) staining for 20 min, and excess stain was rinsed until no background color remained. The plates were dried, and images were taken using an optical microscope. The number of colonies was enumerated.

### Tumor sphere assay

Tumor sphere formation was performed using ultra-low attachment plates. Cells were seeded at 500 cells per well in DMEM/F12 (Gibco) containing 1% B27 supplement, 20 ng/mL EGF, and 20 ng/mL bFGF (PeproTech Inc., NJ, USA). The medium was refreshed every 2 d. After 14 d, images of tumor spheres were taken, and the number of spheres (over 50 μm in diameter) was enumerated.

### Cell cycle analysis

Cells were digested with trypsin, collected, and washed twice with phosphate-buffered saline (PBS). Cells were fixed overnight in 70% pre-chilled ethanol at 4℃. After fixation and washing, the cells were stained with 50 µg/mL RNase A (Sigma-Aldrich) and 100 µg/mL propidium iodide in the dark. The cell cycle distribution was analyzed by flow cytometry (BD Biosciences, Franklin Lakes, NJ, USA), and the proportions of cells in G0/G1, S, and G2/M phases were analyzed using FlowJo software.

### Assessment of multinucleated cells

The treated cells seeded on coverslips were fixed and permeabilized with 0.1% Triton X-100 for 10 min. Nuclei were stained with 4’, 6-diamidino-2-phenylindole (DAPI; Solarbio) for 5 min, and coverslips were mounted with anti-fade medium. Nuclear morphology was observed in 10 random fields under a fluorescence microscope, and the multinucleated cells were enumerated.

### Immunofluorescence staining

Cells were cultured on coverslips, fixed, and permeabilized. After blocking with 5% BSA for 1 h, cells were incubated with alpha-Tubulin antibody (1:200, Abcam) overnight at 4℃. The next day, cells were washed and incubated with fluorophore-conjugated IgG (1:500, Invitrogen) for 1 h in the dark. After staining, spindle formation was observed under a fluorescence microscope.

### Fluorescence co-localization assay

Fluorescence assays were employed to analyze the co-localization of TET2 and BACH1 in cells. H1299 or H1975 cells were seeded at 3 × 10^4^ cells per well on sterilized glass slides (12-well plates). After reaching 50–70% confluence, the medium was discarded, and cells were washed, followed by fixation, permeabilization, and BSA blocking for 1 h. Primary antibodies (anti-TET2, Abcam; anti-BACH1, CST) were added and incubated overnight at 4℃. The following day, cells were washed and probed with Alexa Fluor 488- or Alexa Fluor 594-conjugated IgG (Invitrogen) for 1 h. After further washing, the cells underwent nuclear staining using DAPI and were then mounted. The distribution of fluorescent signals in the cells was analyzed under a confocal microscope. Fluorescence images of TET2 and BACH1 were collected in the 488 nm and 594 nm channels, respectively, while the DAPI channel was used to visualize the nuclei. The overlapping regions of the two fluorescent signals were analyzed using ImageJ software.

### Luciferase reporter assays

A luciferase reporter construct containing the PRC1 promoter region was generated and co-transfected with a Renilla luciferase reporter construct in a 4:1 ratio into 293T cells (24-well plate, 50% confluence). After 24 h, the cells were loaded to serum-free medium and treated with different concentrations of Bobcat339. In another separate experiment, the reporter construct was co-transfected with the BACH1 overexpression plasmid into 293T cells. After 48 h, cells were collected, and luciferase activity was determined utilizing the dual-luciferase reporter assay system (Promega Corporation, Madison, WI, USA).

### Bisulfite sequencing of PRC1 promoter under TET2 regulation

To investigate the DNA methylation status of the PRC1 promoter at single-base resolution, genomic DNA was isolated from H1299 cells stably administered sh-TET2 or control shRNA using the DNeasy Blood & Tissue Kit (#69506, Qiagen Company, Düsseldorf, Hilden, Germany). A total of 1 µg genomic DNA was subjected to bisulfite conversion using the EZ DNA Methylation-Gold Kit (#D5005, Zymo Research, Irvine, CA, USA) following the manufacturer’s protocol. Bisulfite-specific primers targeting the PRC1 promoter region (− 500 bp to + 200 bp relative to TSS) were designed using MethPrimer. PCR products were amplified using ZymoTaq PreMix, gel-purified, and cloned into the pMD19-T vector (#6013, Takara, Takara Holdings Inc., Kyoto, Japan). At least 10 individual colonies per condition were randomly selected for Sanger sequencing (TSINGKE Biotech, Beijing, China). The methylation status of each CpG was visualized as lollipop plots and quantified using QUMA software (http://quma.cdb.riken.jp).

### Chromatin Immunoprecipitation (ChIP)-qPCR

To examine whether recruitment of TET2 and BACH1 to the PRC1 promoter is dynamically regulated by epigenetic stress, H1299 cells were treated with increasing concentrations of 5-Azacytidine (0, 0.5, 1, or 5 µM; Sigma-Aldrich, #A2385) for 24 h. After treatment, cells (1 × 10^7^) were cross-linked with 1% formaldehyde for 10 min at room temperature and quenched with 125 mM glycine. ChIP-qPCR was then performed following the protocols of the EZ-ChIP kit (Millipore Corp., Billerica, MA, USA). Cells were soaked in 1% formaldehyde for 10 min for DNA-protein crosslinking, followed by neutralization with 125 mM glycine for 5 min. After lysis, DNA was truncated to 200–500 bp fragments utilizing a sonicator (Scientz-II D). Antibodies (anti-TET2 or anti-BACH1, CST) and Protein A/G magnetic beads were then supplemented, and the samples were incubated overnight at 4℃. The next day, the beads were washed, DNA fragments were eluted, and cross-linking was reversed. DNA was purified utilizing a Qiagen DNA purification kit (Qiagen GmbH, Hilden, Germany). qPCR was performed to amplify the PRC1 promoter region, and the abundance of immunoprecipitated DNA was normalized to the input control.

### Co-immunoprecipitation (Co-IP)

Cells were lysed in IP lysis buffer (Solarbio) on ice, followed by centrifugation. The supernatant was harvested and incubated with specific antibodies (anti-TET2 or anti-BACH1, 1:50, CST) and Protein A/G magnetic beads (Thermo Fisher Scientific) overnight at 4℃. The following day, the beads were washed, and the bound proteins were eluted and boiled for 5 min, followed by detection using WB analysis.

### Bioinformatic prediction and prioritization of PRC1 promoter-binding transcription factors

Putative transcription factors (TFs) binding to the PRC1 promoter were identified through two complementary databases. 182 and 241 transcription factors that can bind to the PRC1 promoter from the hTFtarget (http://bioinfo.life.hust.edu.cn/hTFtarget/#!/) and JASPAR (http://jaspar.genereg.net/) databases, respectively. An intersecting analysis led to the identification of 76 intersecting factors. This pool was too large to select a suitable target for subsequent analysis. Subsequently, cut-off values were set for both datasets. For the hTFtarget system, transcription factors with a peak signal greater than 3000 (76 factors) were collected, and for the JASPAR system, transcription factors with a putative binding score greater than 9.2 (214 factors) were collected. BACH1 was finally identified as a promising factor selected for subsequent analysis. Co-IP and fluorescence co-localization assays were performed in H1299 and H1975 cells using anti-TET2 and anti-BACH1 antibodies (as above), followed by secondary antibodies conjugated to Alexa Fluor 488 or 594 (Invitrogen, #A-11034, #A-11037).

### Animal experiments

Male nude mice (6–8 weeks old) and C57BL/6 mice (provided by Anburui Biotechnologies, Fuzhou, Fujian, China) were used for animal experiments. All experiments were approved by the Institutional Animal Care and Use Committee of the First Affiliated Hospital of Soochow University(Approval number: 2024-HSYY-551). For subcutaneous xenograft experiments, H1299 or A549 cells (1 × 10^6^ cells) with stable knockdown or overexpression of PRC1, TET2, or CCNY were mixed with Matrigel and injected subcutaneously into the nude mice (6 mice per group) at the right flank site. Tumor volumes were measured every 7 d utilizing calipers (formula: volume = length × width^2^/2).

To generate isograft tumor models in immunocompetent mice, murine Lewis lung carcinoma cells (3LL) were transduced with a lentiviral construct encoding firefly luciferase (pLenti-EF1α-Luc-Puro, #17477, Addgene) to generate stable luciferase-labeled 3LL-luc cells. Transduction was carried out using standard lentiviral infection protocols with 8 µg/mL polybrene (#H9268, Sigma-Aldrich). Transduced cells were selected using puromycin (2 µg/mL, #A1113803, Thermo Fisher Scientific) for 7 d. Luciferase activity was confirmed via bioluminescence imaging after D-luciferin incubation (150 mg/kg intraperitoneally). For orthotopic transplantation, C57BL/6 male mice (6–8 weeks old, ~ 20–22 g, Anburui Biotech, Fujian, China) were anesthetized using 1.5–2% isoflurane. A small incision was made between the 4th and 5th ribs to expose the left lung lobe. Using a 30-gauge insulin syringe (BD), 1 × 10^6^ 3LL-luc cells in 100 µL of sterile PBS were injected slowly into the left lung parenchyma. Following injection, the chest wall was closed with absorbable sutures, and the skin was sealed with tissue adhesive (Vetbond, 3 M, St. Paul, MN, USA). Mice were monitored until full recovery from anesthesia. Tumor growth was monitored weekly using the IVIS Spectrum imaging system (PerkinElmer, Waltham, MA, USA) after intraperitoneal injection of D-luciferin (150 mg/kg in 100 µL PBS, #LUCK-1G, GoldBio, St. Louis, MO, USA). Bioluminescence images were acquired 10–15 min after injection and quantified using Living Image software. Endpoint tumor burden was confirmed by gross anatomical examination and histological analysis.

For drug treatments, the following compounds were used: TET2 antagonist Bobcat339 (10 mg/kg), BACH1 antagonist HPPE (10 mg/kg), PLK1-IN-10 (10 mg/kg), or the CDK16/CCNY antagonist Rebastinib (10 mg/kg). For the subcutaneous xenograft tumors, drug was administered intra-tumorally from day 7 to day 28, with 50 µL of solution directly injected into the tumor (DMSO/PBS, 1:9). Treatment was performed the other day for 4 weeks. For orthotopic isograft tumors, drugs were injected via the tail vein in 100 µL volumes at the same dose, from day 7 to day 28.

At the experiment endpoint, animals were euthanized with an overdose of 1% nembutal (150 mg/kg, intraperitoneally). Subsequently, the tumor tissues were harvested, weighed, and preserved in 4% formaldehyde (for immunohistochemistry [IHC]) or liquid nitrogen (for molecular analysis). Mice were monitored every 2 d for weight loss and behavioral changes. If weight loss exceeded 20% or adverse behaviors were observed (e.g., reduced activity or fur ruffling), the experiment was terminated early, and data were recorded.

### Tissue microarray (TMA) analysis

TMA experiments were conducted to evaluate the expression of TET2, PRC1, and CCNY in NSCLC and to analyze their relationships with clinical characteristics. The TMAs, provided by a commercial company, included 104 lung cancer patient samples with both cancerous and adjacent tissues (1.5 mm diameter per sample). After drying at 60℃ for 1 h, sections were deparaffinized with xylene (twice, 10 min each), rehydrated with graded alcohols (100%, 95%, 85%, 70%, 5 min each), and washed with distilled water for 2 min.

Antigen retrieval was achieved in citrate buffer (pH 6.0) at 121℃ for 2 min, followed by PBS washes. The samples were exposed to 3% H_2_O_2_ to block endogenous peroxidase activity, washed, and then blocked with 5% BSA. After removing the blocking solution, primary antibodies (anti-TET2, Abcam; anti-phospho-PRC1, CST; anti-CCNY, Abcam) were added for an overnight incubation at 4℃.

The next day, the samples were washed and incubated with HRP-conjugated IgG (ZSGB-Bio Co., Ltd, Beijing, China) at 37℃ for 30 min, followed by color development using DAB. Subsequently, the sections were counterstained with hematoxylin, dehydrated, cleared, and mounted. The TMAs were analyzed under a microscope. H-scores were calculated using the formula: H-score = Σ[percentage of positive cells × intensity grade], and the correlations between concerned genes and the clinical characteristics (e.g., stage, lymph node metastasis, and distant metastasis) were analyzed using Pearson’s correlation analyses. Similar procedures were performed on the tumor sections prepared from mouse xenograft tissues to analyze the staining intensities of Ki67 and cleaved caspase 3 (C-Cas3).

### Statistical analysis

Data were collected from a minimal of six biological replicates and analyzed using SPSS version 22.0 (IBM Corp. Armonk, NY, USA). Measurement data are exhibited as the mean ± standard deviation. Unpaired *t*-tests were applied for comparisons between two groups. When multiple groups were concerned, one-way or two-way analysis of variance was applied, followed by Tukey’s post-hoc analysis. Data are presented as dots and bars. The significance level was set at *P* < 0.05.

## Results

### PRC1 knockdown weakens malignant behavior of NSCLC cells

**Fig. 1:**
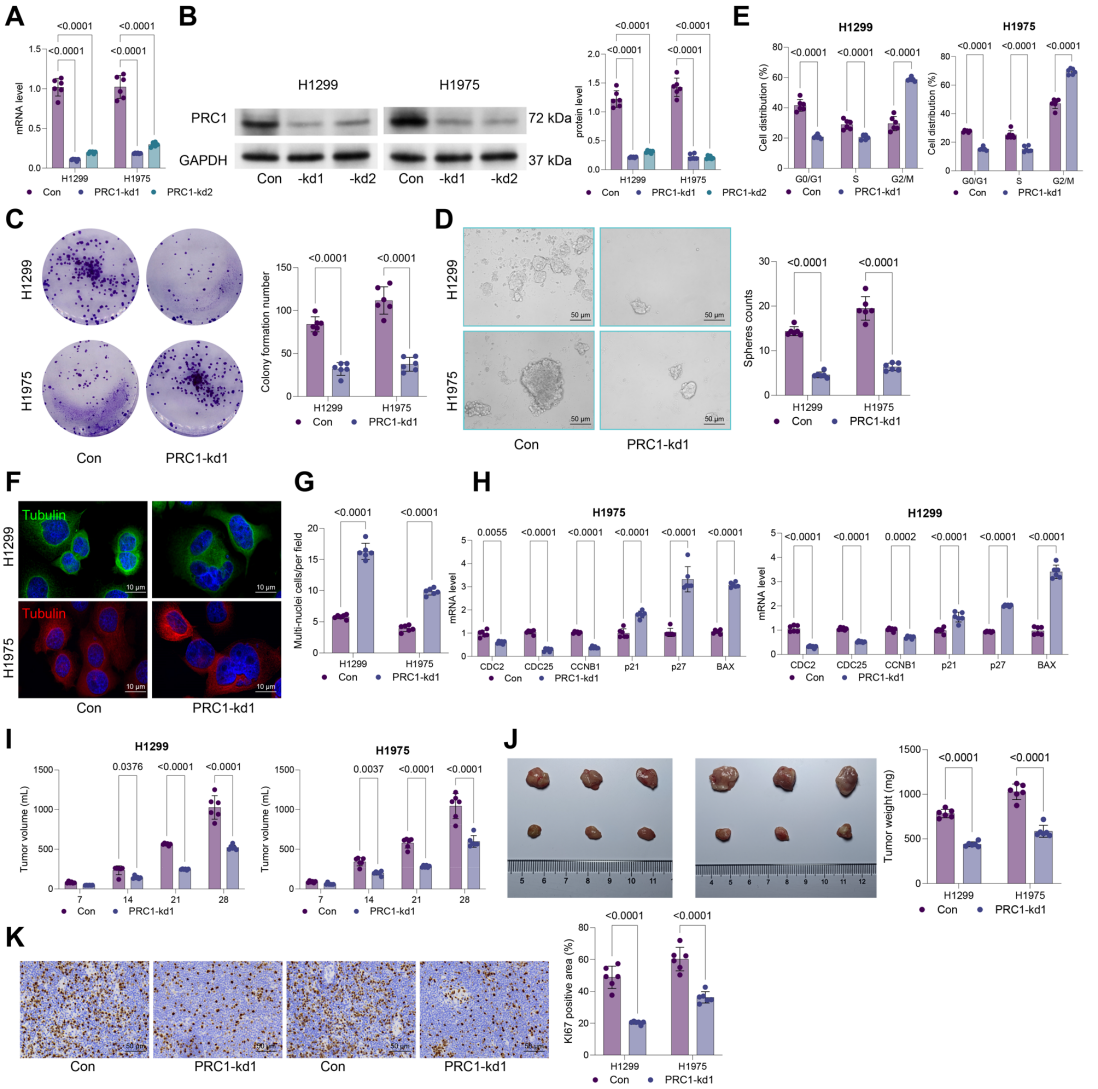
PRC1 knockdown suppresses malignant properties of NSCLC cells. **A**-**B**, mRNA (**A**) and protein (**B**) levels of PRC1 in H1299 and H1975 cells after administration of shRNA targeting PCR1 (PRC1-kd1 and PRC1-kd2) determined using qPCR and WB assays. H1299 and H1975 cells transfected with PRC1-kd1 were collected for subsequent analysis. **C**, colony formation ability of transfected cells analyzed using colony formation assay; **D**, sphere formation ability of transfected cells analyzed using sphere formation assay; **E**, cell cycle distribution in cells analyzed by flow cytometry; **F**, immunofluorescence staining of alpha-Tubulin to analyze mitotic spindle formation; **G**, number of multinucleated cells analyzed by DAPI staining; **H**, mRNA expression of key cell cycle regulators (CDC25A, CCNB1, and CDC2) and apoptosis-related genes (p27, p21, and BAX) determined using qPCR analysis. H1299 or H1975 cells with stable PRC1 knockdown were injected into nude mice. **I**, volume of xenograft tumors by week; **J**, representative images and weight of xenograft tumors after day 28; **K**, positive staining of KI67 in tumor tissues determined using IHC. Cellular experiments were repeated 6 times. For animal experiments, each group contained 6 mice. Data are presented as dots and bars. The significance level was set at *P* < 0.05

We have previously established PRC1 as a promising risk factor linking to LUAD progression [7]. To further investigates its role, we assessed PRC1 expression in several cell lines. As anticipated, the NSCLC cell lines (H1299, H1975, A549, and Calu-3) exhibited higher mRNA and protein levels compared to BEAS-2B cells (Fig. S1A-B). The H1299 and H1975 cells, which show the highest levels or PRC1, were selected for loss-of-function assays. PRC1 specific knockdown was induced in these two cell lines with shRNAs (PRC1-kd1 and PRC1-kd2). The PRC1-kd1 demonstrating greater knocking down efficiency was applied in subsequent experiments (Fig. 1A-B). The PRC1 knockdown markedly suppressed colony formation (Fig. 1C) and spheroid formation (Fig. 1D) abilities of H1299 and H1975 cells in vitro.

Given PRC1’s key role in spindle formation during cell division, we hypothesized that its dysregulation might affect cell cycle progression. Cell cycle analysis confirmed that PRC1 knockdown led to G2/M phase arrest in both H1299 and H1975 cells, accompanied by significant spindle formation abnormalities and a notably increase in multinucleated cells (Fig. 1E-G). Further analysis showed that the PRC1 knockdown significantly downregulated cell cycle regulators CDC25A, CCNB1, and CDC2, while upregulating apoptosis-related genes, including p21, p27, and BAX (Fig. 1H). Furthermore, H1299 and H1975 cells with stable PRC1 knockdown were implanted into nude mice subcutaneously for in vivo experiments. The tumorigenic activity of both cell lines was substantially inhibited upon PRC1 knockdown (Fig. 1I-K), with a marked decrease in the number of KI67-positive cells within the tumors (Fig. 1L).

**Fig. 2:**
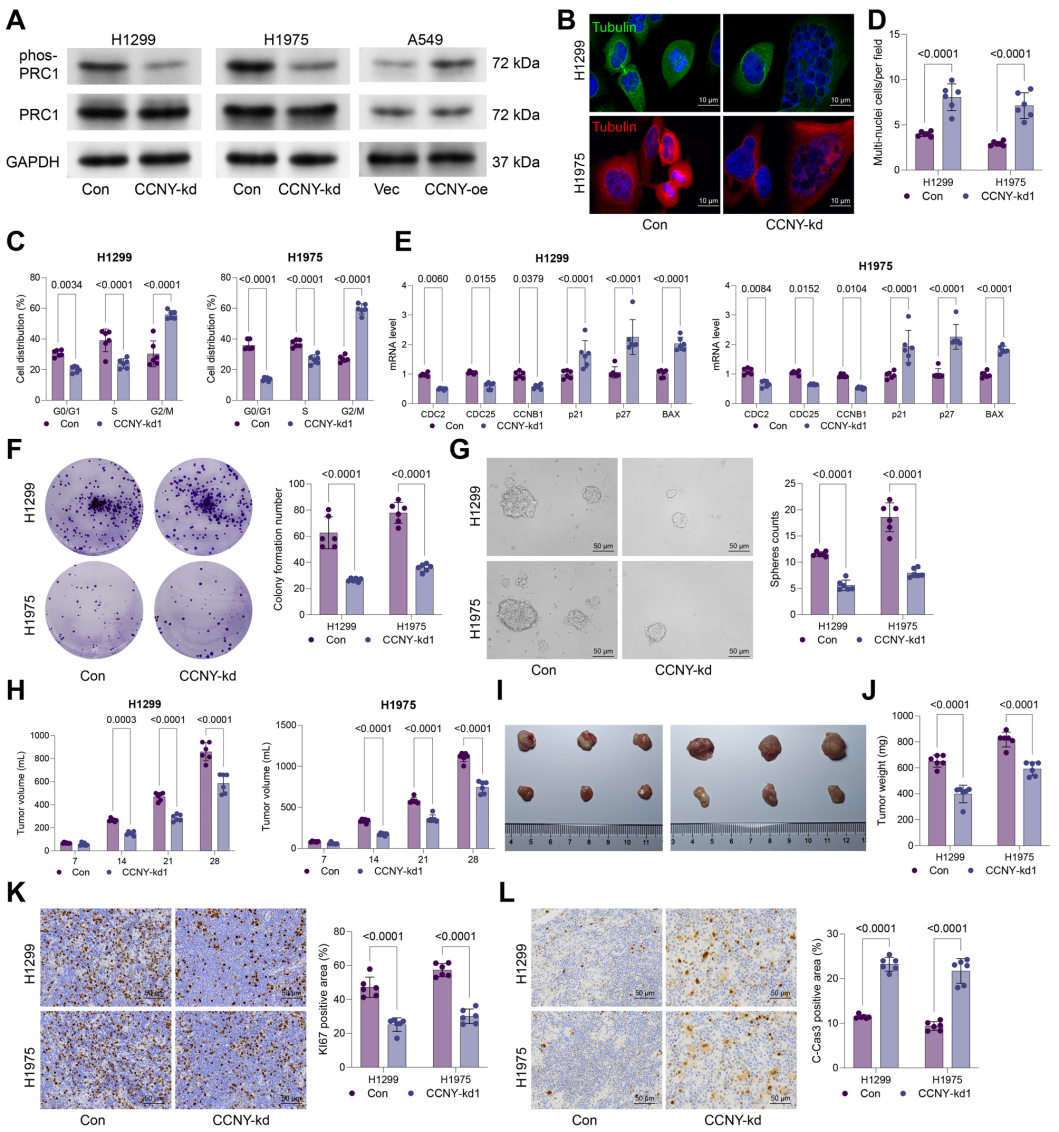
CCNY knockdown suppresses malignant properties of NSCLC cells. H1299 and H1975 cells after administration of shRNA targeting CCNY. **A**, phosphorylation and protein levels of PRC1 in cells determined using WB analysis; **B**, immunofluorescence staining of alpha-Tubulin to analyze mitotic spindle formation; **C**, cell cycle distribution in cells analyzed by flow cytometry; **D**, number of multinucleated cells analyzed by DAPI staining; **E**, mRNA expression of key cell cycle regulators (CDC25A, CCNB1, and CDC2) and apoptosis-related genes (p27, p21, and BAX) determined using qPCR analysis; **F**, colony formation ability of cells analyzed using colony formation assay; **G**, sphere formation ability of cells analyzed using sphere formation assay. H1299 or H1975 cells with stable CCNY knockdown were injected into nude mice. **H**, volume of xenograft tumors by week; **I**-**J**, representative images (**I**) and weight (**J**) of xenograft tumors after day 28; **K**-**L**, positive staining of KI67 (**K**) and C-Cas3 (**L**) in tumor tissues determined using IHC. Cellular experiments were repeated 6 times. For animal experiments, each group contained 6 mice. Data are presented as dots and bars. The significance level was set at *P* < 0.05

In contrast, overexpression of PRC1 in A549 cells — which initially expressed the lowest level of PRC1 among the several studied NSCLC cell lines—led to a substantial increase in colony formation and spheroid formation abilities (Fig. S1C-F). Meanwhile, the expression of CDC25A, CCNB1, and CDC2 was enhanced while the expression of p21, p27, and BAX was reduced in A549 cells overexpressing PRC1 (Fig. S1G). These results highlight the critical role of PRC1 in promoting malignant progression of NSCLC cells. Furthermore, we generated H1299 cells with PRC1^ko^ using the CRISPR-Cas9 technique. Similarly, the PRC1 knockout in cells substantially reduced the colony formation (Fig S2A), increased G2/M cell cycle arrest (Fig. 2B), reduced sphere formation (Fig S2C), and increased the number of multinucleated cells (Fig S2D). Not surprisingly, these cells showed no levels of PRC1 phosphorylation (Fig S2E).

### CCNY enhances PRC1 phosphorylation and promotes cell cycle progression

Previous research has shown that CCNY interacts with CDK16 to facilitate the phosphorylation of PRC1 and promote cytokinesis [16]. To examine whether this cascade functions in NSCLC cells, we induced CCNY knockdown in H1299 and H1975 cell lines (Fig. S3A). This treatment significantly reduced in PRC1 phosphorylation levels (Fig. 2A), thus reducing spindle formation (Fig. 2B), inducing G2/M phase cell cycle arrest (Fig. 2C), and increasing the number of multinucleated cells (Fig. 2D). Similar to PRC1-kd, the CCNY knockdown reduced the expression of CDC2, CDC25, and CCNB1 while enhanced expression of p21, p27, and BAX in H1299 and H1975 cells (Fig. 2E). The colony and sphere formation capacities of the cells were substantially suppressed upon CCNY knockdown as well (Fig. 2F-G). As anticipated, the in vivo experiments validated that knockdown of CCNY in H1299 and H1975 cells reduced their tumorigenic activity in the nude mice (Fig. 2H-J), accompanied by decreased expression of KI67 while increased expression of C-Cas3 in tumor tissues (Fig. 2K-L).

Conversely, overexpression of CCNY in A549 cells (Fig. S3B) promoted cell cycle progression (Fig. S3C). This procedure augmented colony and sphere formation capacities of cells (Fig. S3D-E). Inverse trends were also found in terms of the expression of CDC2, CDC25, CCNB1, p21, p27, and BAX following CCNY upregulation (Fig S3F). Existing literature has shown that CDK16 modulates tyrosine phosphorylation of PRC1 at the T481 site in breast cancer cells [16, 25]. In this study, we predicted potential phosphorylation sites in PRC1 using the NetPhos 3.1 software, with seven sites predicted (Table S1). Among these, T429A, S431, and T495A had the highest predicted scores. To confirm the specific sites involved in PRC1 phosphorylation, we constructed PRC1 (T429A, S431A, and T495A) variants and had them transfected into PRC1-knockdown cells. Among these, the T429A variant had no significant effect on the growth of PRC1-knockdown cells, whereas the WT and the other two variants significantly promoted cell growth (Fig S4A-D). Additionally, the PRC1 phosphorylation degree was increased in cells loaded with WT, S431A, or T495A variants, phenomenon not observed in those loaded with the T429A variant (Fig S4E). Similarly, compared to the WT variant, administration of the T429A variant did not significantly affect the growth and PRC1 phosphorylation in H1299 cells with PRC1 knockout (Fig S5A-E). This ample evidence suggests that PRC1 phosphorylation at T429 site is significant for PRC1 activation and its oncogenic role in NSCLC.

To substantiate whether CCNY regulates cell cycle progression by modulating PRC1 phosphorylation at T429 site, A549 cells overexpressing PRC1 were exposed to the CDK16/CCNY antagonist NVP-2. This treatment significantly reduced PRC1 phosphorylation in A549 cells (Fig. S6A) and increased the number of multinucleated cells (Fig. S6B), along with a marked decrease in cell proliferation (Fig. S6C-D). Furthermore, the expression of downstream cell cycle regulators was significantly reduced by NVP-2, while apoptotic gene expression was upregulated (Fig. S6E-F). Additionally, PRC1^ko^ H1299 cells were transfected with CCNY-oe or empty control. Nevertheless, the CCNY overexpression in cells failed to induce PRC1 phosphorylation in cells loaded with the T429A variant, though increased phosphorylation was observed in cells administered the WT variant (Fig S6G). Together, these findings suggest that CCNY promotes PRC1 phosphorylation at the at T429 site, which in turn facilitates spindle formation, accelerates cell cycle progression, and supports NSCLC cell growth.

### Treatment with Reb reduces PRC1 phosphorylation and reduces growth of NSCLC cells

**Fig. 3:**
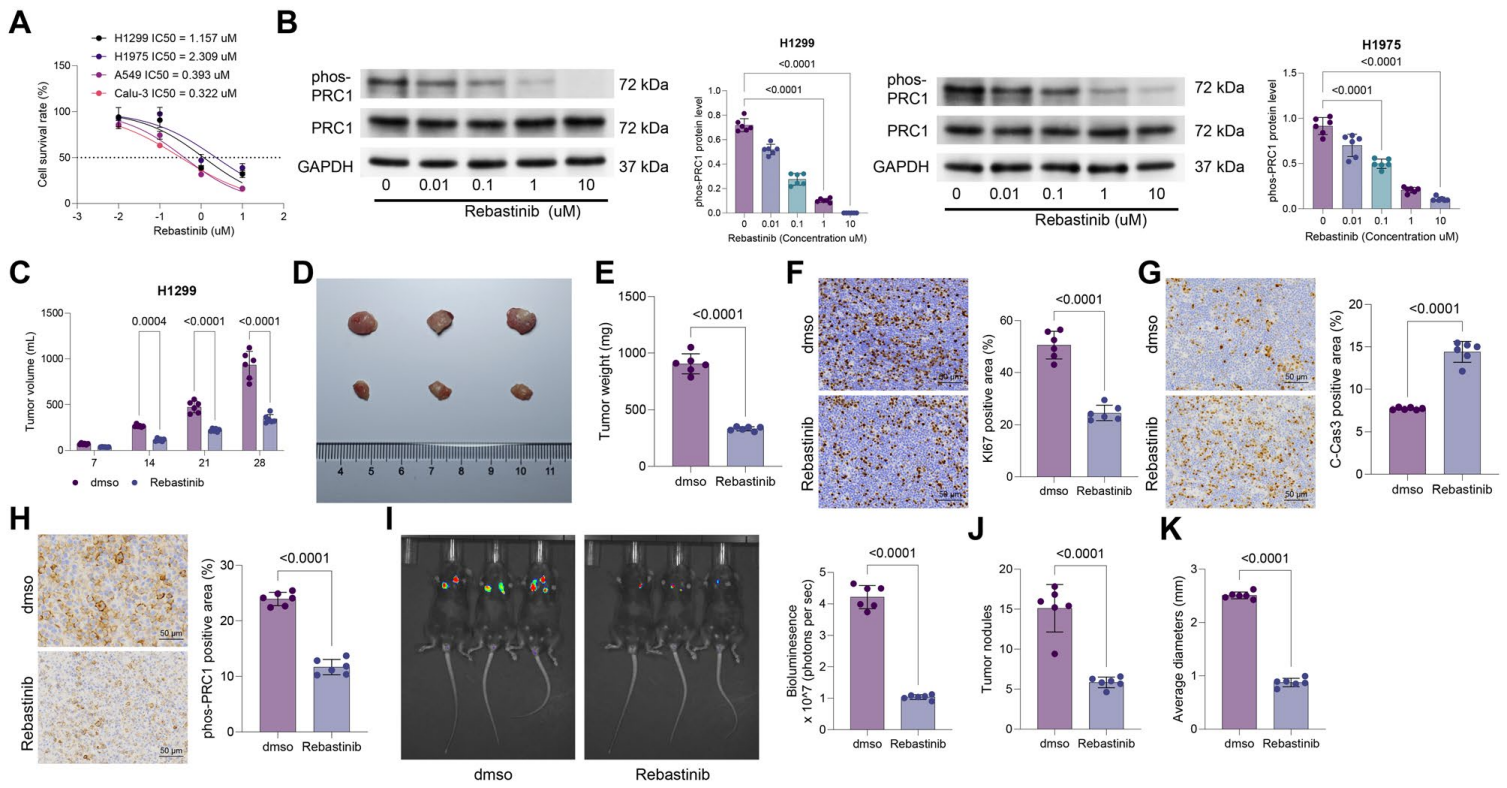
Treatment with Reb reduces PRC1 phosphorylation and reduces growth of NSCLC cells. **A**, H1299, H1975, A549, and Calu-3 cells were exposed to ascending concentrations of Reb (0, 0.1, 1, 10, and 100 µM), followed by viability assessment to evaluate IC50 of Reb in each cell type; **B**, phosphorylation of PRC1 in H1299 and H1975 cells after treatment of different concentrations of Reb (0, 0.1, 1, 10, and 100 µM) determined by WB analysis. H1299 cells were implanted into nude mice subcutaneously, followed by intra-tumoral administration of Reb after day 7. **C**, volume of xenograft tumors by week; **D**-**E**, representative images (**D**) and weight (**E**) of xenograft tumors after day 28; **F**-**H**, positive staining of KI67 (**F**), C-Cas3 (**G**), and phos-PRC1 (**H**) in tumor tissues determined using IHC. Another orthotopic isograft tumor model was generated by injecting mouse 3LL lung cancer cells, labeled with luciferase, into the lung of C57 mice, followed by Reb administration from day 7 to day 28. I, the bioluminescence intensity in mice analyzed by the IVIS; **J**-**K**, number (**J**) and average size (**K**) of the orthotopic tumors after day 35. Cellular experiments were repeated 6 times. For animal experiments, each group contained 6 mice. Data are presented as dots and bars. The significance level was set at *P* < 0.05.

Reb has been identified as an anti-tumor drug and an antagonist of CDK16 that functions by disrupting the CCNY-CDK16 interaction [26]. To assess the therapeutic potential of Reb in NSCLC, we treated the NSCLC cell lines with Reb at different doses. H1299 and H1975 cells with higher PRC1 expression showed increased resistance to Reb treatment, marked by increased Reb IC50 values in these cell lines (Fig. 3A). Furthermore, the Reb treatment resulted in a dose-dependent reduction in PRC1 phosphorylation in H1299 and H1975 cells (Fig. 3B). Parallel findings were observed in vivo. In subcutaneous xenograft tumor models in nude mice formed by H1299 cells, the intra-tumoral administration of Reb significantly suppressed tumor growth, reduced the staining intensity of KI67 and phos-PRC1, and enhanced the staining intensity of C-Cas3 (Fig. 3C-H).

To better mimic the tumor growth and immune microenvironment, we transfected mouse Lewis lung cancer cells (3LL) with luciferase and had them injected into the lungs of immunocompetent C57 mice. After 7 d, the mice were administered Reb via tail vein injection until day 28. This Reb administration significantly reduced the bioluminescence intensity in mice (Fig. 3I), indicating a slowdown in tumor growth. At the end point, both the number and size of tumor lesions in the lung of Reb-treated mice were significantly reduced (Fig. 3J-K).

### TET2 reduces DNA methylation of PRC1 and activates its transcription by interacting with BACH1

**Fig. 4:**
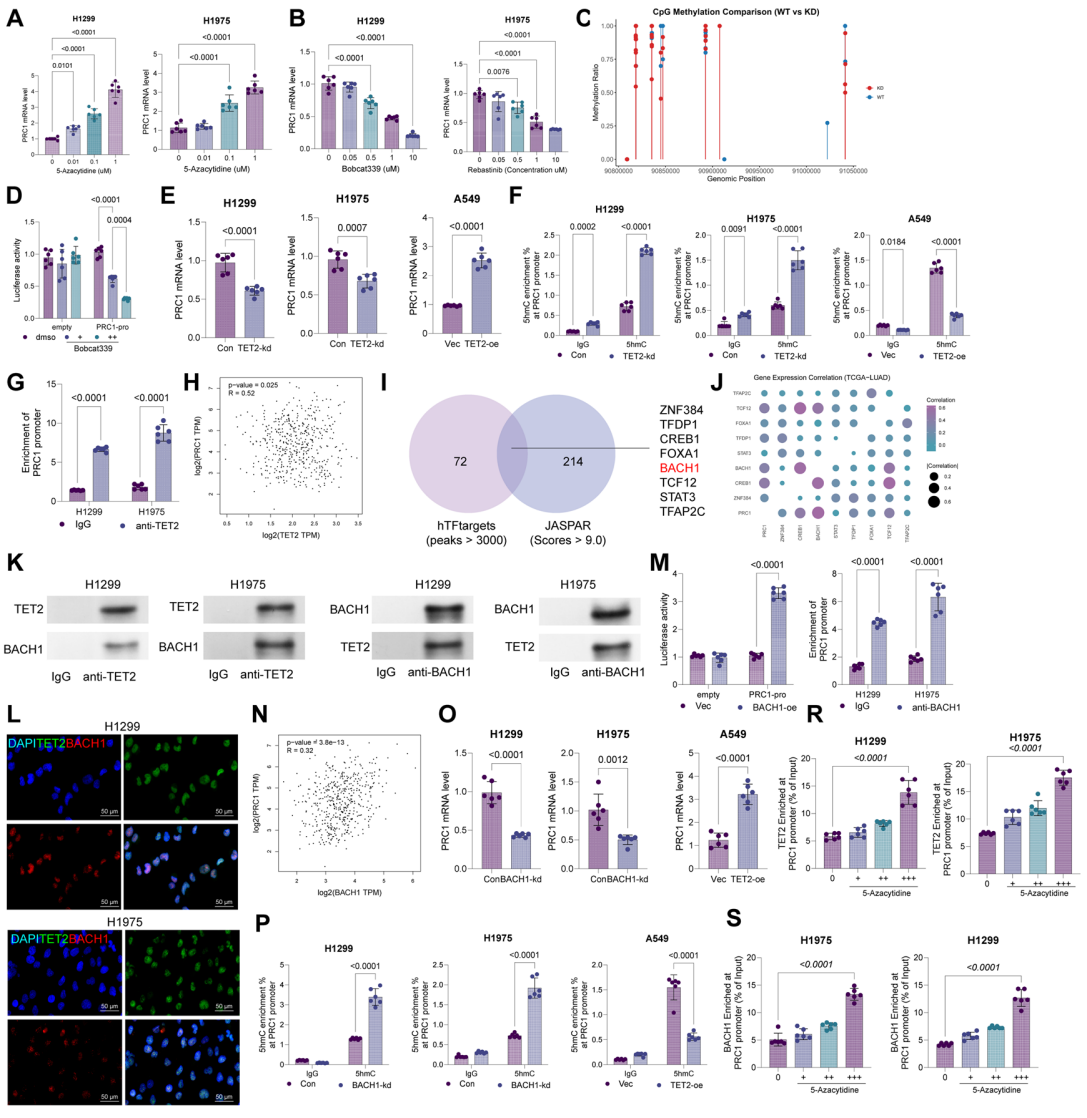
TET2 reduces DNA methylation of PRC1 and activates its transcription by interacting with BACH1. **A**, PRC1 mRNA expression in H1299 and H1975 cells after treatment with different concentrations of 5-Azacytidine determined by qPCR analysis; **B**, PRC1 mRNA expression in H1299 and H1975 cells after treatment with different concentrations of Bobcat339 determined by qPCR analysis; **C**, PRC1 promoter methylation in TET-kd H1299 cells determined using bisulfite sequencing analysis; **D**, luciferase activity of the reporter vector containing PRC1 promoter in 293T cells treated with Bobcat339; **E**, PRC1 mRNA expression in H1299 and H1975 cells with TET2 knockdown, or in A549 cells with TET2 overexpression, determined by qPCR analysis; **F**, the 5hmC enrichment at the PRC1 promoter region in transfected H1299, H1975, and A549 cells determined by hMeDIP-qPCR; **G**, binding between TET2 and the PRC1 promoter analyzed using ChIP assay; **H**, Pearson’s correlation analysis of the TET2 and PRC1 expression, based on the TCGA-LUAD data; **I**, BACH1 identified as an intersecting transcription factor that binds to PRC1 promoter according to hTFtarget and JASPAR databases; **J**, the correlation between PRC1 and ZNF384, CREB1, BACH1, STAT3, TFDP1, FOXA1, TCF12, and TFAP2C in the TCGA-LUAD dataset analyzed using R correlation analyses; **K**, interaction between TET2 and BACH1 in H1299 and H1975 cells examined by the Co-IP assays; **L**, binding between TET2 and BACH1 in H1299 and H1975 cells verified using immunofluorescence staining; **M**, luciferase activity of the reporter vector containing PRC1 promoter in 293T cells transfected with BACH1-oe; **N**, binding between BACH1 and the PRC1 promoter analyzed using ChIP assay; **O**, Pearson’s correlation analysis of the BACH1 and PRC1 expression, based on the TCGA-LUAD data; **P**, PRC1 mRNA expression in H1299 and H1975 cells with BACH1 knockdown, or in A549 cells with BACH1 overexpression, determined by qPCR analysis; **Q**, the 5hmC enrichment at the PRC1 promoter region in transfected H1299, H1975, and A549 cells determined by hMeDIP-qPCR. R-S, H1299 cells were treated with 5-Aza in graded concentrations, followed by ChIP-qPCR analyses using anti-TET2 or anti-BACH1. Cellular experiments were repeated 6 times. Data are presented as dots and bars. The significance level was set at *P* < 0.05

Our previous research indicated a negative correlation between PRC1 expression and DNA methylation in LUAD [7]. To further verify whether PRC1 is modulated by DNA methylation, we treated H1299 and H1975 cells with 5-Aza, a DNA methyltransferase. Notably, the 5-Aza treatment resulted in a dose-dependent increase in PRC1 mRNA in cells (Fig. 4A). In contrast, treatment with Bobcat339, a TET-specific antagonist, dose-dependently reduced PRC1 mRNA levels (Fig. 4B). Additionally, we extracted genomic DNA from TET2-kd H1299 cells for bisulfite sequencing and found that the methylation levels in the PRC1 promoter segment region were significantly increased in TET2-kd cells (Fig. 4C). This evidence suggests PRC1 expression is regulated by TET2-mediated promoter demethylation.

To verify this result, we generated a luciferase reporter construct containing the PRC1 promoter and had it loaded into 293T cells. Upon treatment with Bobcat339, we found a significant, dose-dependent reduction in luciferase activity (Fig. 4D). We induced TET2 knockdown in H1299 and H1975 cells (Fig. S7A-B). This procedure reduced PRC1 mRNA expression in cells while enhancing CpG methylation (Fig. 4E-F). By contrast, overexpression of TET2 in A549 cells increased PRC1 expression and reduced promoter methylation (Fig. 4E-F, Fig. S7A-B). Further investigation using ChIP experiments revealed that anti-TET2 enriched more PRC1 promoter fragments compared to IgG (Fig. 4G). Additionally, in TCGA-LUAD data, PRC1 expression showed a positive correlation with TET2 levels (Fig. 4H). These observations support that TET2 plays a critical role in regulating PRC1 expression.

However, it is unlikely that a single demethylase alone can regulate gene expression. Subsequently, we explored TFs that can bind to the PRC1 promoter using hTFtarget and JASPAR databases. For the hTFtarget system, transcription factors with a peak signal greater than 3000 (76 factors) were collected, and for the JASPAR system, transcription factors with a putative binding score greater than 9.2 (214 factors) were collected. The intersecting analysis of these two datasets yielded 8 shared factors: ZNF384, CREB1, BACH1, STAT3, TFDP1, FOXA1, TCF12, and TFAP2C. Expression correlation analysis using TCGA-LUAD data via GEPIA2 (http://gepia2.cancer-pku.cn) revealed that BACH1 showed the strongest positive correlation with PRC1 (*R* = 0.43, *p* < 0.001). Based on this, BACH1 was selected for further validation. Co-IP experiments confirmed the interaction between TET2 and BACH1 (Fig. 4K), a finding further supported by fluorescence co-localization experiments (Fig. 4L).

To explore whether BACH1 regulates PRC1 expression, a BACH1 overexpression plasmid was transfected into 293T cells containing luciferase reporter vector containing the PRC1 promoter. The BACH1 overexpression enhanced luciferase activity in cells (Fig. 4M). Furthermore, ChIP experiments confirmed that BACH1 bound to the PRC1 promoter (Fig. 4N). In the TCGA-LUAD dataset, BACH1 presented a positive correlation with PRC1 expression (Fig. 4O). Knockdown of BACH1 in H1299 and H1975 cells caused a reduction in PRC1 expression and an increase in CpG methylation at the PRC1 promoter, with opposite effects observed upon BACH1 overexpression in A549 cells (Fig. 4P-Q, Fig. S7C-D). Furthermore, the H1299 cells treated with a gradient concentration of 5-Aza were harvested for ChIP-qPCR assays. It was found that as the dose of 5-Aza increased, the binding of TET2 and BACH1 to the PRC1 promoter was significantly increased. Additionally, the BACH1 binding to the PRC1 promoter was also increased with the increasing dose of 5-Aza (Fig. 4R-S). This might be attributed to that 5-Aza induces chromatin remodeling and increased promoter accessibility, which may promote the enrichment of various epigenetic regulatory factors, including TET2.

### PRC1 enhances cell cycle progression and growth of NSCLC cells in the presence of TET2 or BACH1 knockdown

**Fig. 5:**
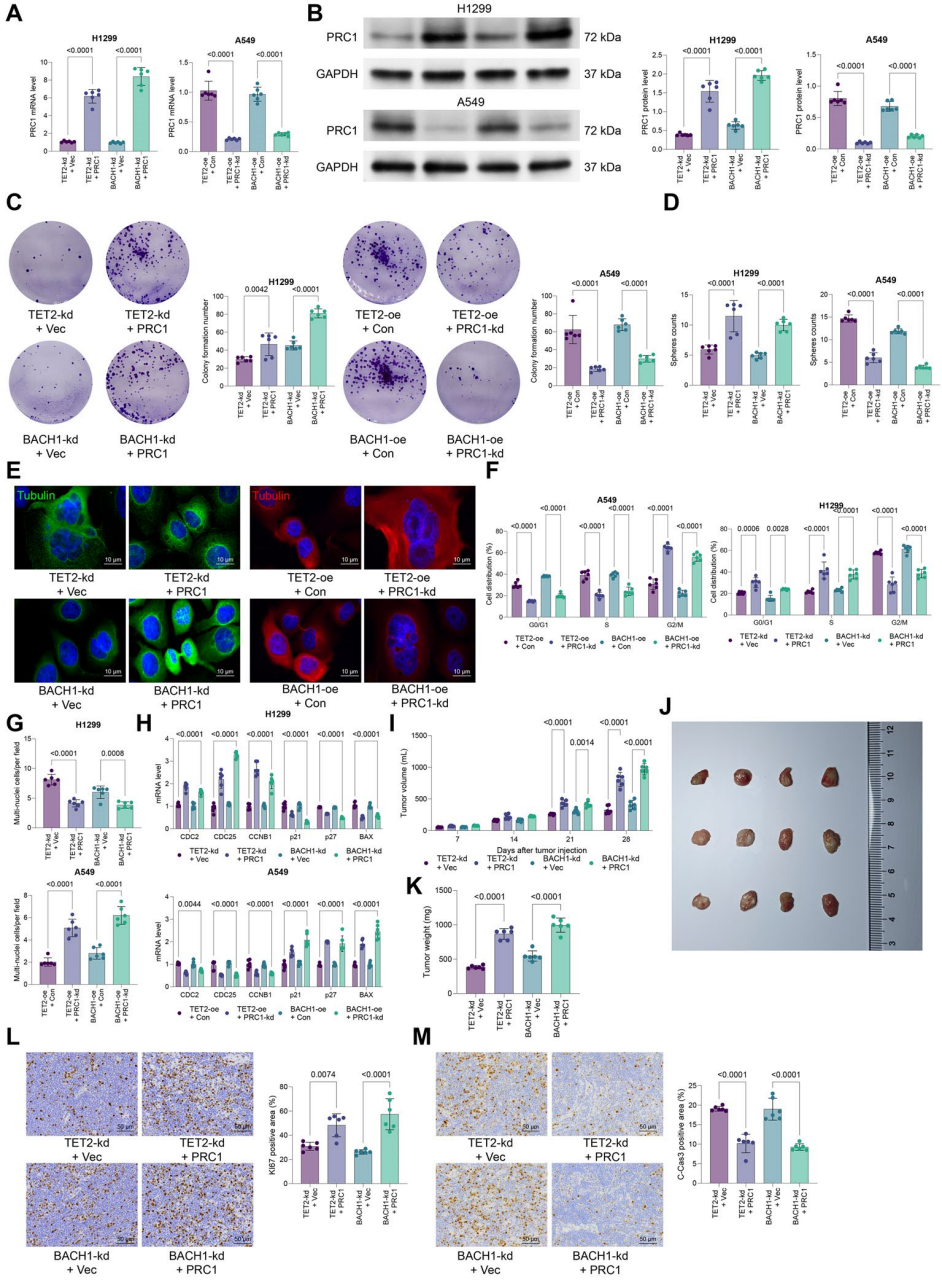
PRC1 enhances cell cycle progression and growth of NSCLC cells in the presence of TET2 or BACH1 knockdown. H1299 cells with stable TET2 or BACH1 knockdown were administered PRC1 overexpression plasmid, while A549 cells with TET2 or BACH1 overexpression were additionally transfected with PRC1-kd. A-B, mRNA (**A**) and protein (**B**) levels of PRC1 in transfected cells analyzed using qPCR and WB analyses. **C**, colony formation ability of transfected cells analyzed using colony formation assay; **D**, sphere formation ability of transfected cells analyzed using sphere formation assay; **E**, immunofluorescence staining of alpha-Tubulin to analyze mitotic spindle formation; **F**, cell cycle distribution in transfected cells analyzed by flow cytometry; **G**, number of multinucleated cells analyzed by DAPI staining; **H**, mRNA expression of key cell cycle regulators (CDC25A, CCNB1, and CDC2) and apoptosis-related genes (p27, p21, and BAX) determined using qPCR analysis. H1299 cells stably transfected with TET2-kd or BACH1-kd were further administered PRC1 overexpression plasmid. Stably transfected cells were injected into nude mice to generate subcutaneous xenograft tumors. **I**, volume of xenograft tumors by week; **J**-**K**, representative images (**J**) and weight (**K**) of xenograft tumors after day 28; **L**-**M**, positive staining of KI67 (**L**) and C-Cas3 (**M**) in tumor tissues determined using IHC. Cellular experiments were repeated 6 times. For animal experiments, each group contained 6 mice. Data are presented as dots and bars. The significance level was set at *P* < 0.05

To further analyze the effect of the TET2-BACH1-PRC1 cascade in malignant properties of NSCLC cells, the H1299 cells with stable TET2 or BACH1 knockdown were administered PRC1 overexpression plasmid. In contrast, A549 cells with TET2 or BACH1 overexpression were additionally transfected with PRC1-kd. The effective gene interference was verified by qPCR and WB analyses (Fig. 5A-B). The results showed that PRC1 restoration, in the presence of TET2 or BACH1 knockdown, significantly promoted growth (Fig. 5C-D), spindle formation and cell cycle progression in H1299 cells in vitro (Fig. 5E-H). By contrast, PRC1 knockdown in A549 cells led to reverse trends (Fig. 5C-H). The PRC1 upregulation following TET2 or BACH1 knockdown also enhanced the tumorigenic capacity of in H1299 cells in the nude mice (Fig. 5I-M).

### Targeting the TET2-BACH1-PRC1 reduces NSCLC suppression

**Fig. 6:**
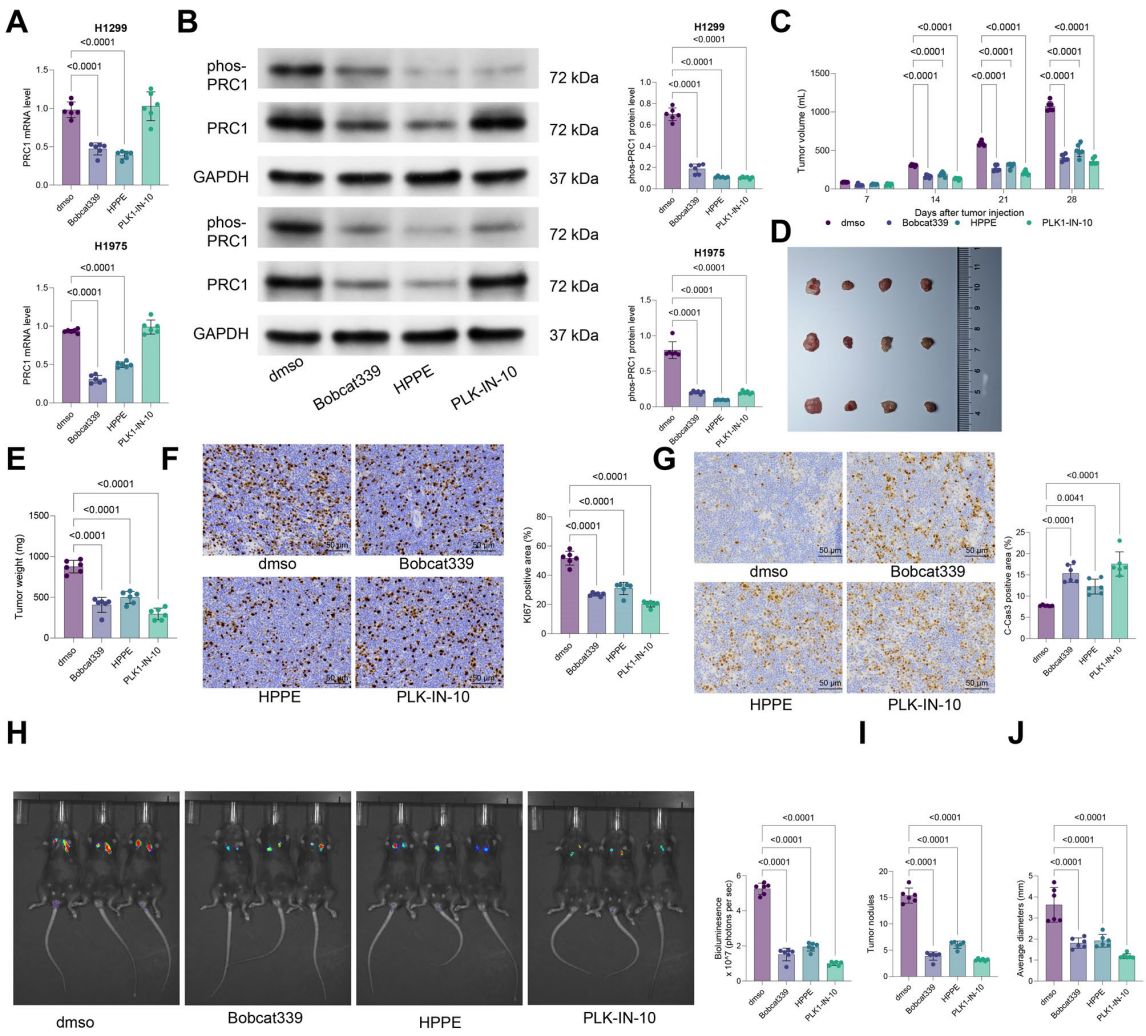
Targeting the TET2-BACH1-PRC1 reduces NSCLC suppression. The H1299 and H1975 cells were exposed to the TET2 antagonist Bobcat339, the BACH1 antagonist HPPE, or the PRC1 antagonist PLK1-IN-10. **A**, mRNA expression of PRC1 in treated cells determined using qPCR analysis; **B**, protein and phosphorylation levels of PRC1 in treated cells analyzed by WB analysis. H1299 cells were injected into nude mice subcutaneously to generate xenograft tumors, followed by intra-tumoral administration of Bobcat339, HPPE, or PLK1-IN-10, starting from day 7. **C**, volume of xenograft tumors by week; **D**-**E**, representative images (**D**) and weight (**E**) of xenograft tumors after day 28; **F**-**G**, positive staining of KI67 (**F**) and C-Cas3 (**G**) in tumor tissues determined using IHC. Another orthotopic isograft tumor model was generated by injecting mouse 3LL lung cancer cells, labeled with luciferase, into the lung of C57 mice, followed by tail vein administration of Bobcat339, HPPE, or PLK1-IN-10, from day 7 to day 28. H, the bioluminescence intensity in mice analyzed by the IVIS; **J**-**K**, number (**J**) and average size (**K**) of the orthotopic tumors after day 28. Cellular experiments were repeated 6 times. For animal experiments, each group contained 6 mice. Data are presented as dot and bars. The significance level was set at *P* < 0.05

To further analyze the function of the TET2-BACH1-PRC1 axis in NSCLC progression, the H1299 and H1975 cells were exposed to the TET2 antagonist Bobcat339, the BACH1 antagonist HPPE, or PLK1-IN-10, an antagonist targets PLK1 to limit the PLK1-PRC1 interaction. Treatment with either Bobcat339 or HPPE substantially reduced the PRC1 mRNA and phosphorylation levels in both cell lines, while PLK1-IN-10 treatment blocked PRC1 phosphorylation without altering its mRNA expression (Fig. 6A-B).

In subcutaneous xenograft tumors formed by H1299 cells in nude mice, the intra-tumoral administration of Bobcat339, HPPE, or PLK1-IN-10 significantly suppressed H1299 cell growth in vivo (Fig. 6C-E), with a marked reduction in KI67 staining intensity and an increase in C-Cas3 staining intensity in tumor tissues (Fig. 6F-G). Similar trends were observed in the orthotopic isograft tumor model, where the treatment of Bobcat339, HPPE, or PLK1-IN-10 markedly reduced the bioluminescence intensity of labeled 3LL cells in the mouse lung (Fig. 6H), accompanied to decreased number and size of tumor burdens (Fig. 6I-J).

### Prognostic values of PRC1, CCNY, and TET2 in NSCLC patients

**Fig. 7:**
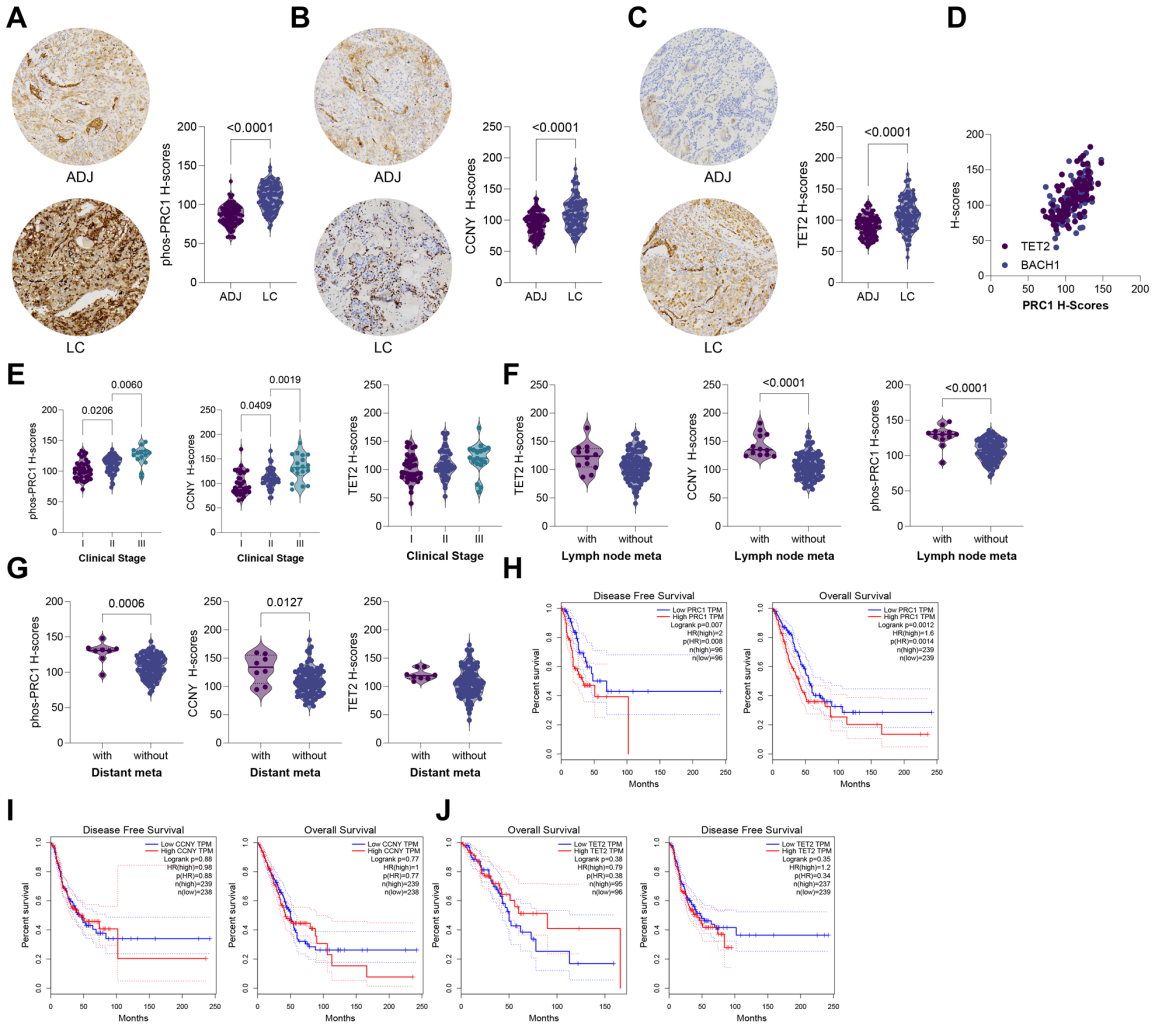
Prognostic values of PRC1, CCNY, and TET2 in NSCLC patients. **A**-**C**, staining intensities of phosphorylated PRC1 (**A**), CCNY (**B**), and TET2 (**C**) in cancerous and adjacent normal tissues from 104 NSCLC TMAs; **D**, Pearson’s correlation analyses of the correlations between TET2/CCNY staining and PRC1 staining in tissue microarrays; **E**-**G**, correlations between PRC1, CCNY, or TET2 and clinical stages (**E**), lymph node metastasis (**F**), and distant metastasis (**G**) in TMAs; H-J, correlations between PRC1 (**H**), CCNY (**I**), or TET2 (**J**) and OS and DFS of patients in the TCGA-LUAD database. Data are presented as dots and bars. The significance level was set at *P* < 0.05

To explore the clinical relevance of PRC1, CCNY, and TET2, we analyzed TMAs from 104 NSCLC patients, which included both cancerous and adjacent normal tissues. First, significantly higher staining intensities of phosphorylated PRC1, CCNY, and TET2 were detected in tumor tissues compared to normal tissues (Fig. 7A-C). Additionally, a strong positive correlation was found between the staining intensities of phosphorylated PRC1 and TET2 or CCNY (Fig. 7D).

Further analysis of the correlations between these molecules and patient characteristics demonstrated that the staining intensities of phosphorylated PRC1 and CCNY were linked to advanced stages, lymph node metastasis, and distant metastasis in patients. However, the staining intensity of TET2 did not show significant correlation with these clinical characteristics (Fig. 7E-G). Nevertheless, data from the TCGA-LUAD dataset revealed that PRC1, CCNY, and TET2 were linked to both shorter overall survival (OS) and progression-free survival (PFS) in patients (Fig. 7H-J).

## Discussion

**Fig. 8:**
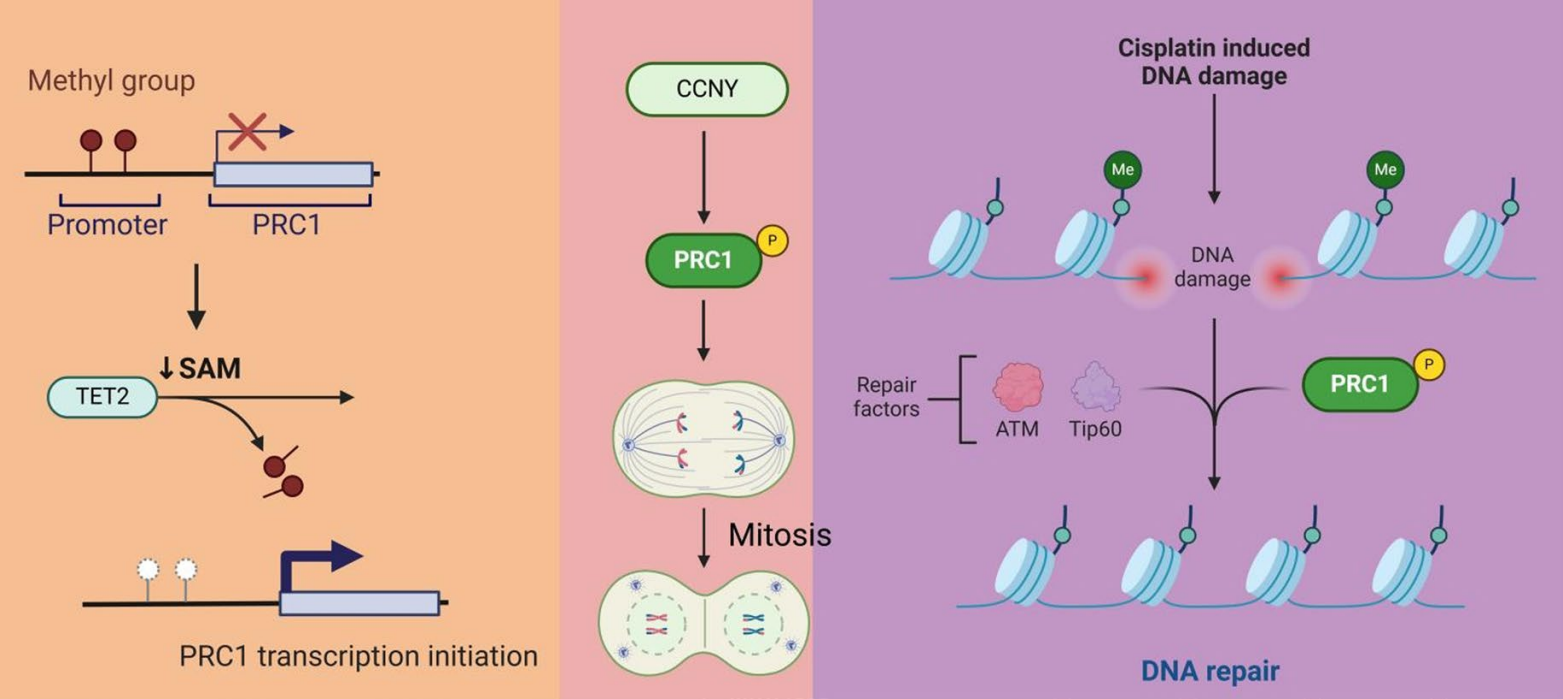
The oncogenic role of PRC1 and its regulatory mechanisms in NSCLC. PRC1 drives cell division and promotes tumor growth. The TET2/BACH1 signaling cascade and CCNY are key regulators of PRC1 overactivation in NSCLC. TET2 and BACH1 promote PRC1 DNA methylation and gene transcription, while CCNY enhances PRC1 phosphorylation.

Building upon our previous findings, which identified PRC1 as a risk factor in lung cancer with low DNA methylation level, this study demonstrates that TET2 is responsible for the demethylation and transcriptional activation of PRC1 in NSCLC cells by interacting with BACH1. Additionally, we reveal CCNY as a crucial regulator of PRC1 phosphorylation in the context of NSCLC progression (Fig. 8).

We observed elevated PRC1 expression in NSCLC cells. Silencing PRC1 in these cells resulted in reduced colony and sphere formation, impaired spindle assembly, and disruption of the G2/M cell cycle. These findings are consistent with prior research [14, 27, 28]. Jiang et al. previously reported that knockdown of endogenous PRC1 disrupted mitosis and prevented proper cytokinesis, leading to multinucleated cells [8]. Similarly, our study found that PRC1 silencing induced the formation of multinucleated cells. Additionally, PRC1 knockdown significantly decreased the tumorigenic potential of NSCLC cells in nude mice. In line with this, Hanselmann et al. showed that PRC1 deletion in mice reduced lung tumorigenesis caused by the K-RAS and the loss of p53 [15]. Our findings provide further evidence of PRC1’s critical role in driving oncogenic processes during NSCLC progression.

Furthermore, previous research have underscored the importance of the CDK16/CCNY complex in promoting PRC1 phosphorylation [16], which is essential for PRC1 oligomerization and the proper organization of the central spindle [29]. In breast cancer cells, phosphorylation of PRC1 by the CDK16/CCNY complex has been linked to accelerated cell cycle progression and tumor growth [25]. Yue et al. also reported that CCNY knockdown suppressed NSCLC cell proliferation and growth, with the opposite effect observed upon CCNY overexpression [19]. Building on these findings, we investigated whether CCNY regulates the malignant characteristics of NSCLC cells through modulation of PRC1. As anticipated, CCNY knockdown in NSCLC cells reduced PRC1 phosphorylation, leading to G2/M cell cycle arrest, impaired spindle formation, and diminished cell growth both in vitro and in vivo. Moreover, administration of Reb, a specific inhibitor of the CDK16/CCNY complex, successfully suppressed the malignant traits of A549 cells, which had been initially enhanced by PRC1 overexpression. In vivo, Reb treatment reduced the tumorigenic capacity of both human and mouse NSCLC cells in nude and immunocompetent mice, respectively. These results suggest that PRC1 phosphorylation plays a pivotal role in the tumor-promoting effects of CCNY in NSCLC. However, while previous publications suggest that CDK16 promotes tyrosine phosphorylation of PRC1 at the T481 site [16, 25], we constructed several PRC1 (T429A, S431A, and T495A) variants and identified that PRC1 phosphorylation at T429 was indispensable for PRC1 activation and its oncogenic role in NSCLC. While CCNY overexpression restores malignant properties of PRC1^ko^ cells after reintroduction PRC1 WT phosphorylation residues, it failed to rescue cell malignancy in the absence of T429 phosphorylation site.

Our additional findings indicate that treatment with either 5-Azacytidine or Bobcat339 significantly reduced PRC1 expression in NSCLC cells, suggesting that the aberrant upregulation of PRC1 in NSCLC is, at least in part, regulated by TET-mediated DNA demethylation. TET proteins are iron- and α-ketoglutarate-dependent dioxygenases that catalyze the oxidation of 5mC to 5hmC, 5-formylcytosine, and 5-carboxylcytosine, progressively altering the methylation landscape of DNA [30]. Among these, TET2 has emerged as a gene of particular interest, as it is frequently mutated in various cancers [24, 31].

While the loss of TET2 has been linked to the upregulation of the tumor necrosis factor /nuclear factor kappa B signaling pathway, which confers resistance to EGFR-tyrosine kinase inhibitors in NSCLC [32], we observed that TET2 overexpression in NSCLC cells notably increased PRC1 expression through promoter demethylation. This process probably includes the transformation of 5mC into 5hmC, resulting in a more accessible chromatin structure. This, in turn, facilitates the binding of TFs to the DNA, triggering the activation of gene expression [33].

In this study, we identified a critical interaction between TET2 and BACH1, a member of the CNC-bZIP transcription factor family. BACH1 is widely expressed across mammalian tissues and exerts crucial functions in regulating heme homeostasis, oxidative stress responses, and epigenetic modifications [34]. Increased BACH1 expression has been observed in various malignancies, including lung cancer, where it modulates processes such as oxidative stress response, cell cycle regulation, mitosis, and cellular senescence [35]. Importantly, high levels of functional BACH1 have been associated with aggressive cancer phenotypes and poor patient prognosis in lung cancer [36, 37]. Our findings show that TET2 recruits BACH1 to the PRC1 promoter region, where BACH1 then facilitates the transcriptional activation of PRC1. Silencing either TET2 or BACH1 impaired the tumorigenic potential of NSCLC cells in animal models. This evidence underscores the importance of the TET2-BACH1 interaction in regulating the demethylation and transcriptional activation of PRC1, highlighting its critical role in NSCLC progression.

Additional clinical validation using TMAs revealed that CCNY, TET2, and phosphorylated PRC1were significantly upregulated in tumor tissues. While TET2 did not show a specific correlation with advanced TNM stages of NSCLC patients, unlike CCNY and phos-PRC1, bioinformatics analyses indicated that all three molecules were associated with OS and DFS. These results suggest that PRC1, TET2, and CCNY could serve as valuable prognostic biomarkers and potential therapeutic targets in NSCLC.

In summary, this study demonstrates that PRC1 plays a crucial oncogenic role in NSCLC by driving cell division and tumor growth. It highlights the TET2/BACH1 signaling cascade and CCNY as key regulators of PRC1 overactivation in NSCLC. TET2 and BACH1 promote PRC1 DNA demethylation and gene transcription, while CCNY enhances PRC1 phosphorylation. These findings offer new insights into the molecular mechanisms underpinning NSCLC progression and suggest novel avenues for targeted therapeutic interventions. However, several limitations must be acknowledged. First, the potential off-target effects of the small-molecule inhibitors used (such as Rebastinib, Bobcat339, HPPE, and PLK1-IN-10) were not fully ruled out. The absence of inactive analog controls or orthogonal inhibitors limits our ability to completely exclude off-target influences. Additionally, for most gene-interference experiments, only a single shRNA was employed in functional assays, which may reduce the overall comprehensiveness of this study. We aim to address these gaps in future investigations.

## Electronic supplementary material

Below is the link to the electronic supplementary material.

Supplementary Material 1

Supplementary Material 2

Supplementary Material 3

Supplementary Material 4

Supplementary Material 5

Supplementary Material 6

Supplementary Material 7

Supplementary Material 8

## Data Availability

No datasets were generated or analysed during the current study.

## Abbreviations

BACH1: BTB domain and CNC homolog 1
BSA: Bovine serum albumin
CCNY: Cyclin Y
CCNY: Cleaved caspase 3
CDKs: Cyclin-dependent kinases
ChIP: Chromatin immunoprecipitation
Co-IP: Co-immunoprecipitation
DAPI: 4’, 6-diamidino-2-phenylindole
IHC: Immunohistochemistry
IVIS: In vivo imaging system
LUAD: Lung adenocarcinoma
NSCLC: Non-small cell lung cancer
PBS: Phosphate-buffered saline
PRC1: Protein regulator of cytokinesis 1
qPCR: Quantitative polymerase chain reaction
shRNA: Short hairpin RNA
TET2: Tet methylcytosine dioxygenase 2
TMA: Tissue microarray
WB: Western blot
5mC: 5-methylcytosine
5hmC: 5-hydroxymethylcytosine
