# Supplementary figures and images for: CCNY-mediated phosphorylation and TET2-BACH1-driven DNA demethylation activate PRC1 to augment NSCLC progression
Source: J Exp Clin Cancer Res. 2025 Jul 15;44:206. doi: 10.1186/s13046-025-03472-x (PMC12261648; doi:10.1186/s13046-025-03472-x)

**A**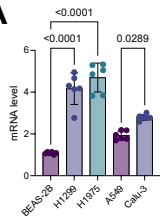**B**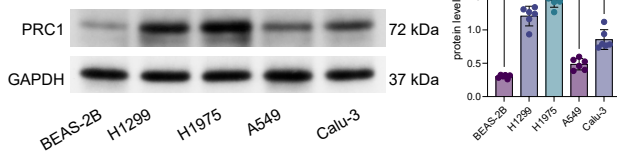**C**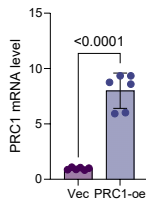**D**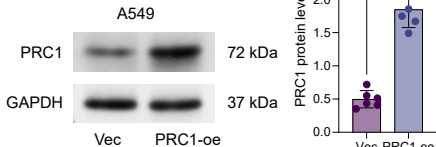**E**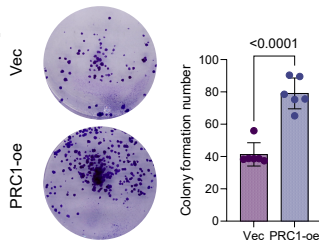**F**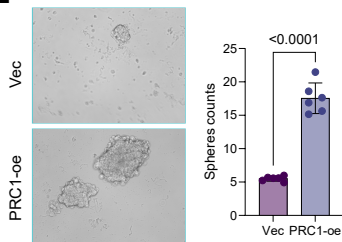**G**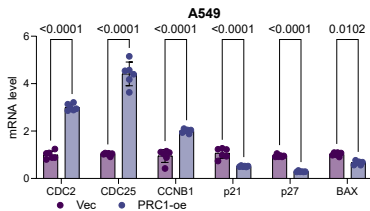

Supplement: Supplementary file 1 — Supplementary Material 1 [file 13046_2025_3472_MOESM1_ESM.pdf]

**A**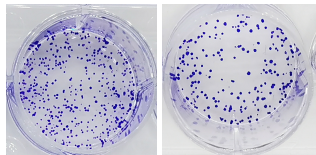

WT

PRC1<sup>ko</sup>**B**

H1299

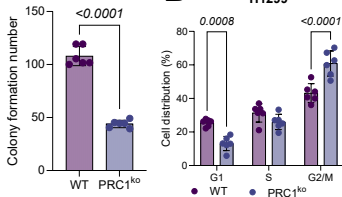**C**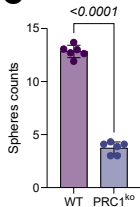**D**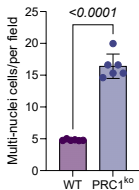**E**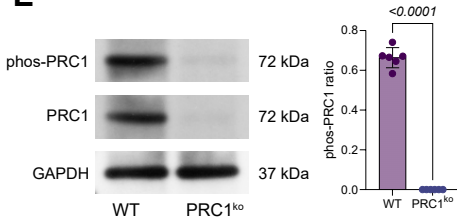

Supplement: Supplementary file 2 — Supplementary Material 2 [file 13046_2025_3472_MOESM2_ESM.pdf]

**A**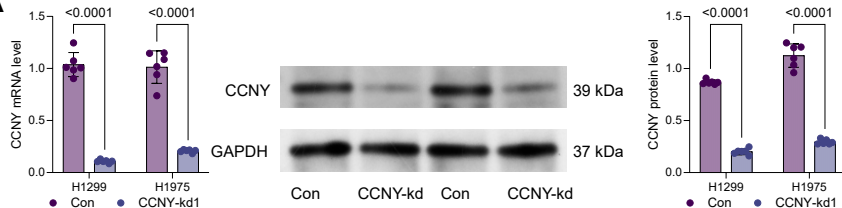**B**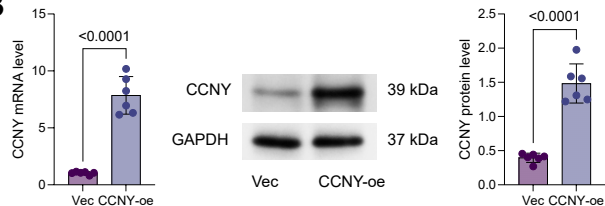**C**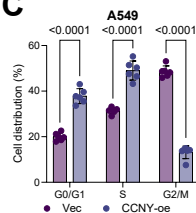**D**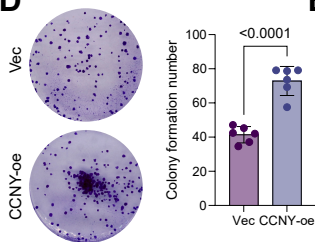**E**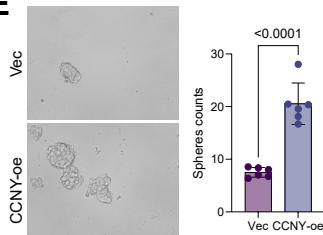**F**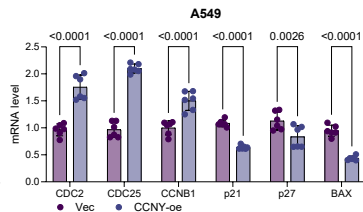

Supplement: Supplementary file 3 — Supplementary Material 3 [file 13046_2025_3472_MOESM3_ESM.pdf]

**A**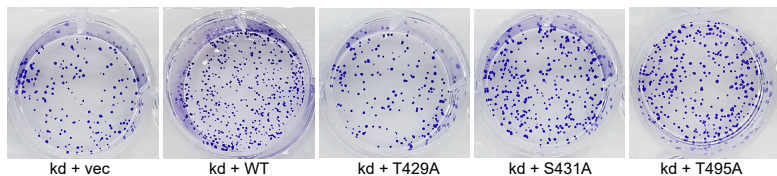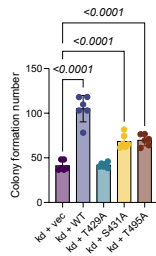**B**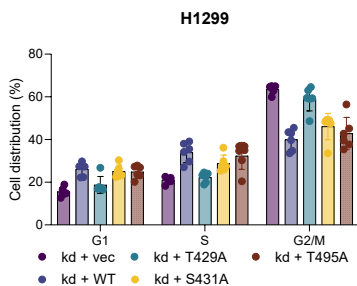**C**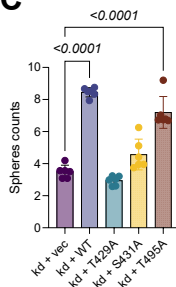**D**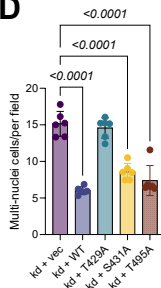**E**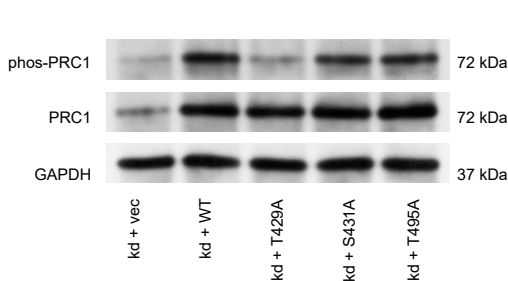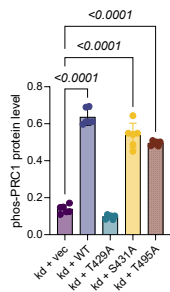

Supplement: Supplementary file 4 — Supplementary Material 4 [file 13046_2025_3472_MOESM4_ESM.pdf]

**A**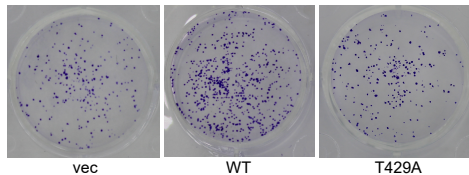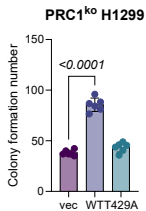**B**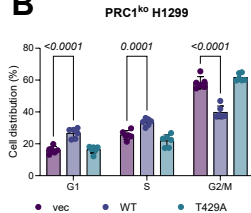**C**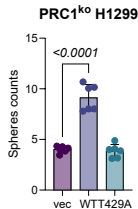**D**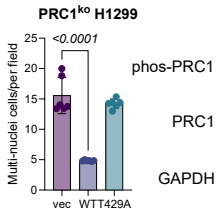**E**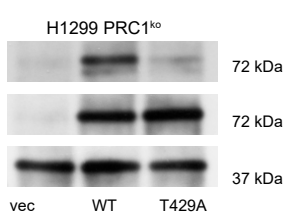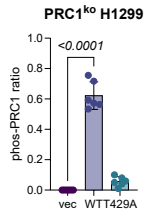

Supplement: Supplementary file 5 — Supplementary Material 5 [file 13046_2025_3472_MOESM5_ESM.pdf]

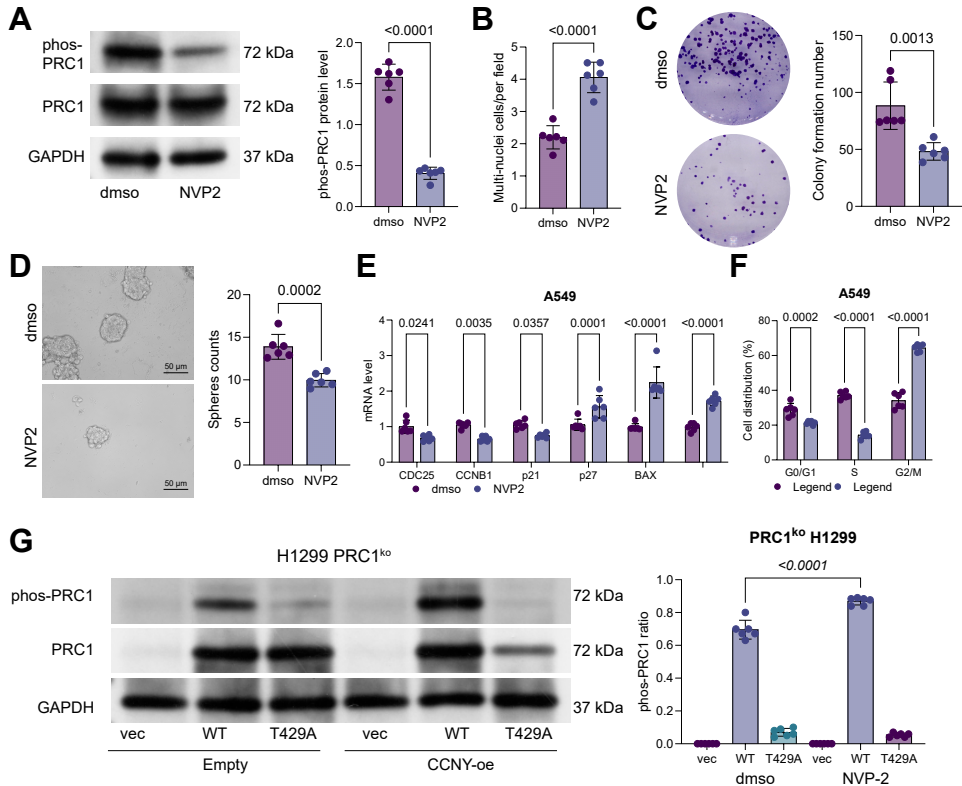

Supplement: Supplementary file 6 — Supplementary Material 6 [file 13046_2025_3472_MOESM6_ESM.pdf]

**A**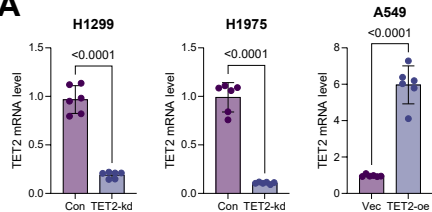**B**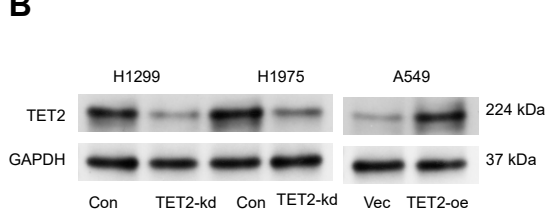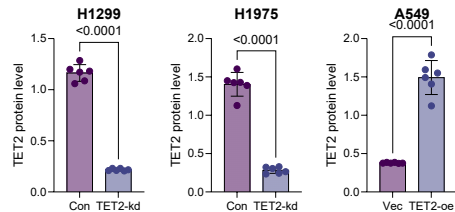**C**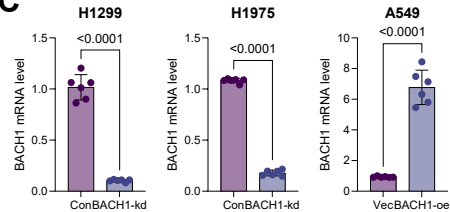**D**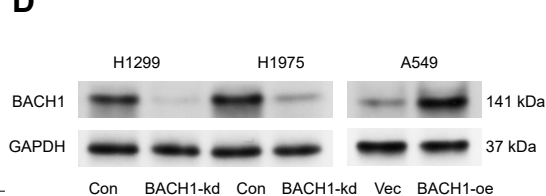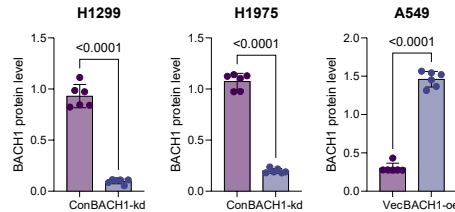

Supplement: Supplementary file 7 — Supplementary Material 7 [file 13046_2025_3472_MOESM7_ESM.pdf]
