# Supplementary material for: CCNY-mediated phosphorylation and TET2-BACH1-driven DNA demethylation activate PRC1 to augment NSCLC progression
Source: J Exp Clin Cancer Res. 2025 Jul 15;44:206. doi: 10.1186/s13046-025-03472-x (PMC12261648; doi:10.1186/s13046-025-03472-x)
Supplement: Supplementary file 8 — Supplementary Material 8 [file 13046_2025_3472_MOESM8_ESM.docx]

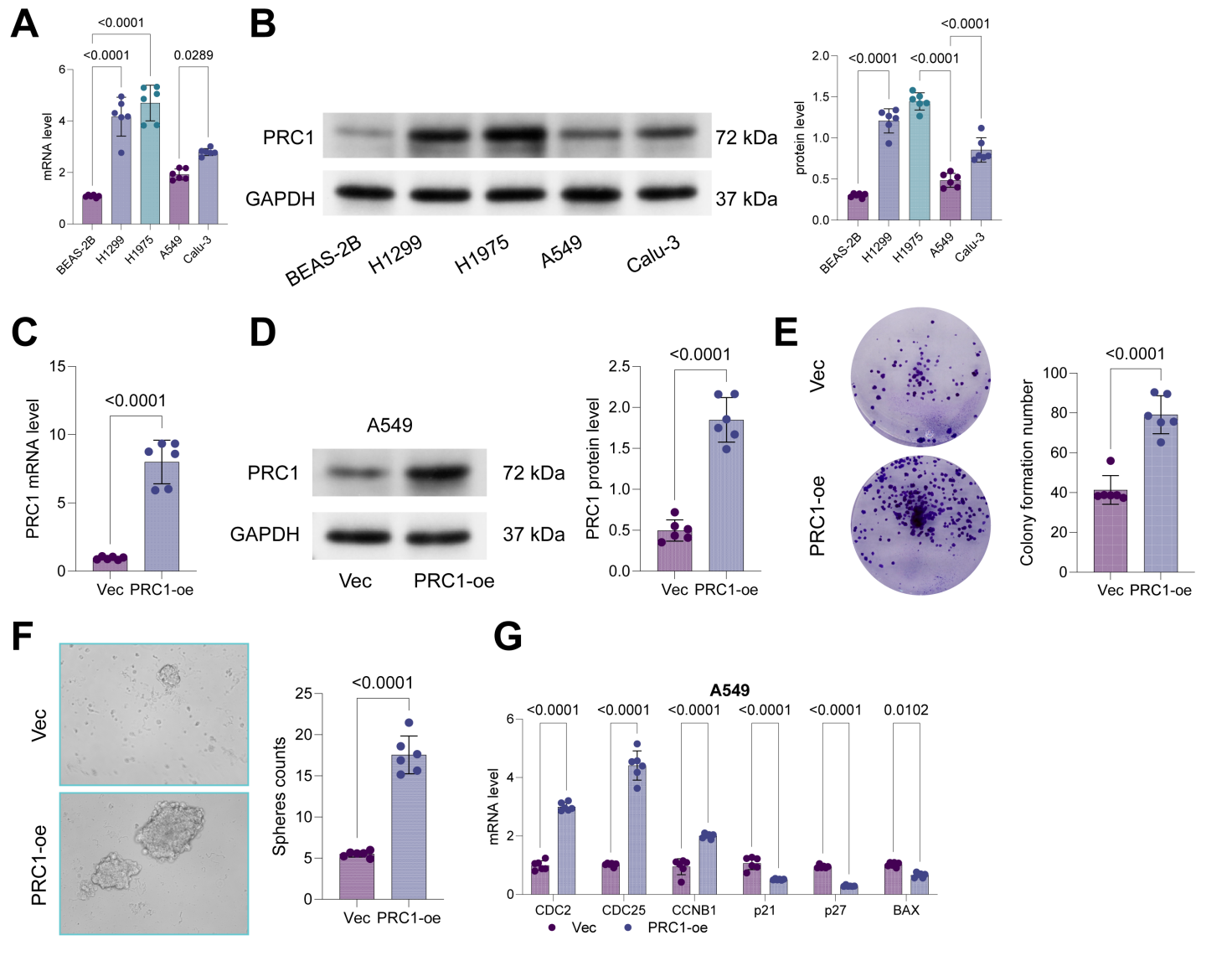


**Fig. S1.** **Expression pattern of PRC1 in NSCLC cell lines and its function in cell growth *in vitro*.** A-B, mRNA (A) and protein (B) levels of PRC1 in NSCLC cell lines (H1299, H1975, A549, and Calu-3) and normal BEAS-2B cells analyzed using qPCR and WB analyses. A549 cells expressing the lowest levels of PRC1 among the several NSCLC cell lines were administered PRC1-overexpression plasmid or a control plasmid. C-D, mRNA (C) and protein (D) levels of PRC1 in transfected cells determined using qPCR and WB analyses; E, colony formation ability of transfected cells analyzed using colony formation assay; F, sphere formation ability of transfected cells analyzed using sphere formation assay; G, mRNA expression of key cell cycle regulators (CDC25A, CCNB1, and CDC2) and apoptosis-related genes (p27, p21, and BAX) determined using qPCR analysis. Cellular experiments were repeated 6 times. Data are presented as dots and bars. The significance level was set at *P* < 0.05.

**
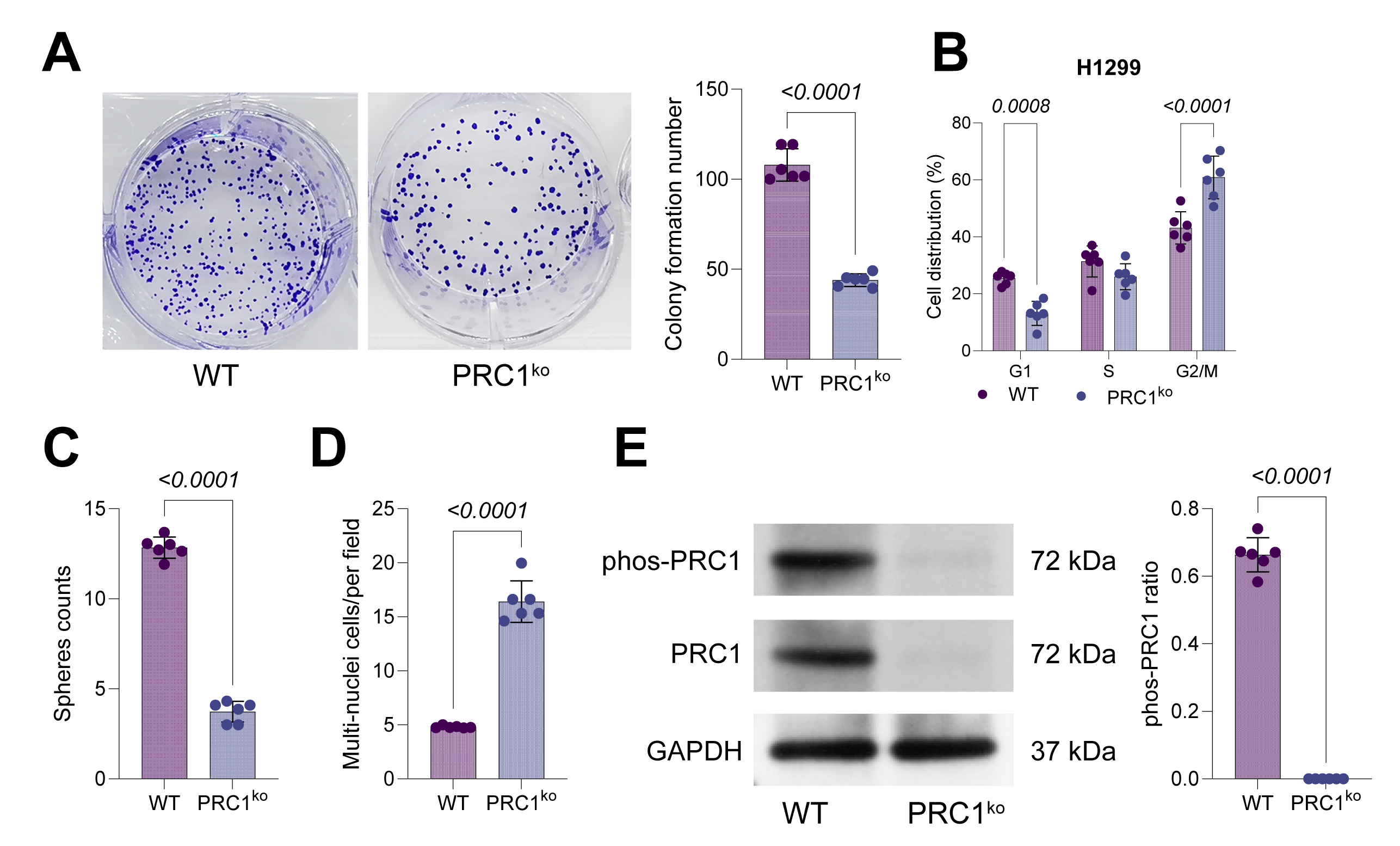
**

**Fig S2.** PRC1 knockout in H1299 cells reduces cell growth. H1299 cells with PRC1 gene knockout were generated using the CRISPR-Cas9 technique. A, colony formation ability of cells analyzed using colony formation assay; B, cell cycle distribution in cells analyzed by flow cytometry; C, sphere formation ability of cells analyzed using sphere formation assay; D, number of multinucleated cells analyzed by DAPI staining; E, phosphorylation of PRC1 in cells determined using WB analysis. Cellular experiments were repeated 6 times. Data are presented as dots and bars. The significance level was set at *P* < 0.05.


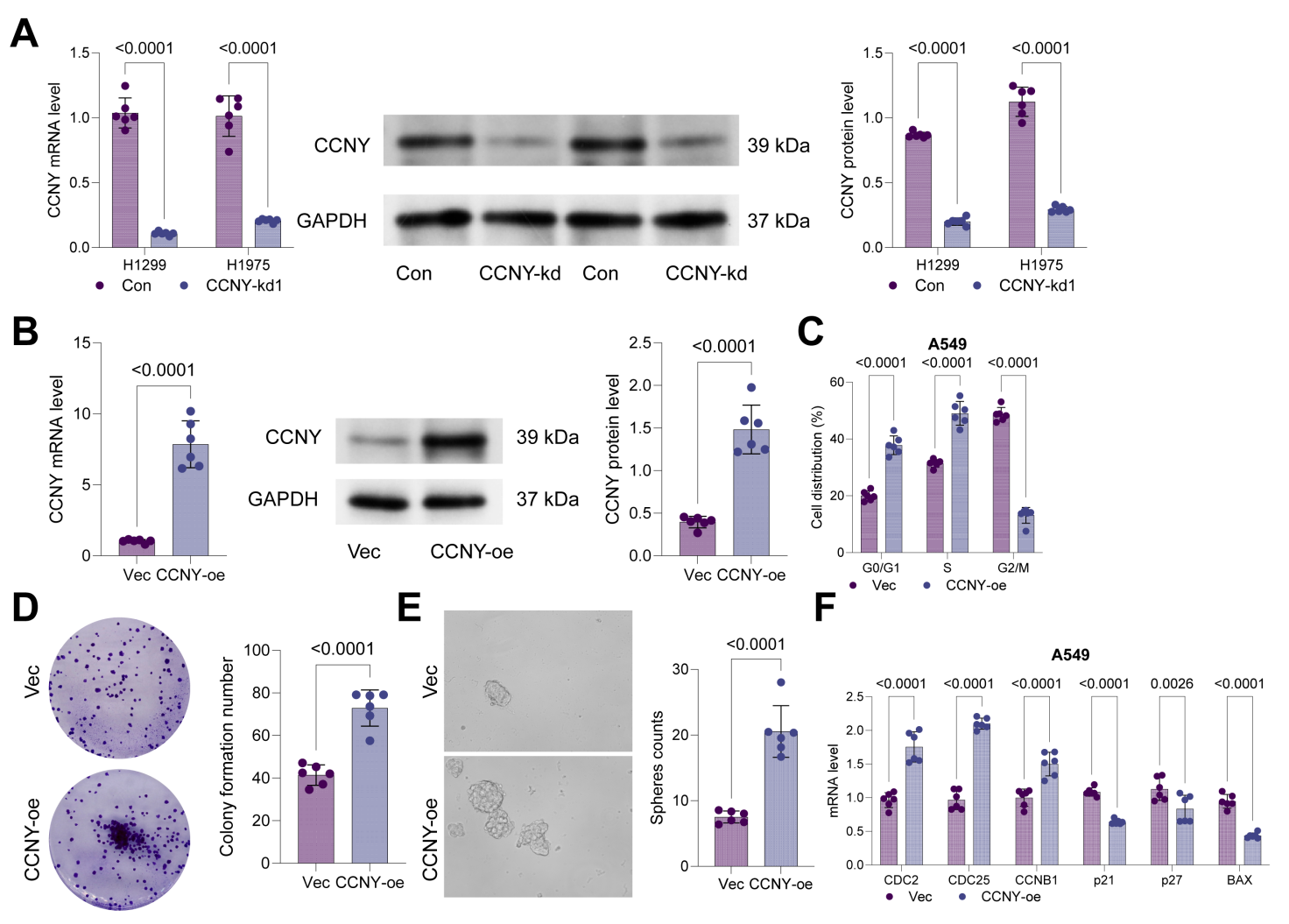


**Fig. S3. Successful gene interference of CCNY in NSCLC cells and its role in the cell properties.** A, mRNA and protein levels of CCNY in H1299 and H1975 cells after transfection of CCNY-kd1 analyzed using qPCR and WB analyses; B, mRNA and protein levels of CCNY in A549 cells after transfection of CCNY-oe determined using qPCR and WB analyses; C, cell cycle distribution in transfected A549 cells analyzed by flow cytometry; D, colony formation ability of transfected A549 analyzed using colony formation assay; E, sphere formation ability of transfected A549 cells analyzed using sphere formation assay; F, mRNA expression of key cell cycle regulators (CDC25A, CCNB1, and CDC2) and apoptosis-related genes (p27, p21, and BAX) in transfected A549 cells determined using qPCR analysis. Cellular experiments were repeated 6 times. Data are presented as dots and bars. The significance level was set at *P* < 0.05.


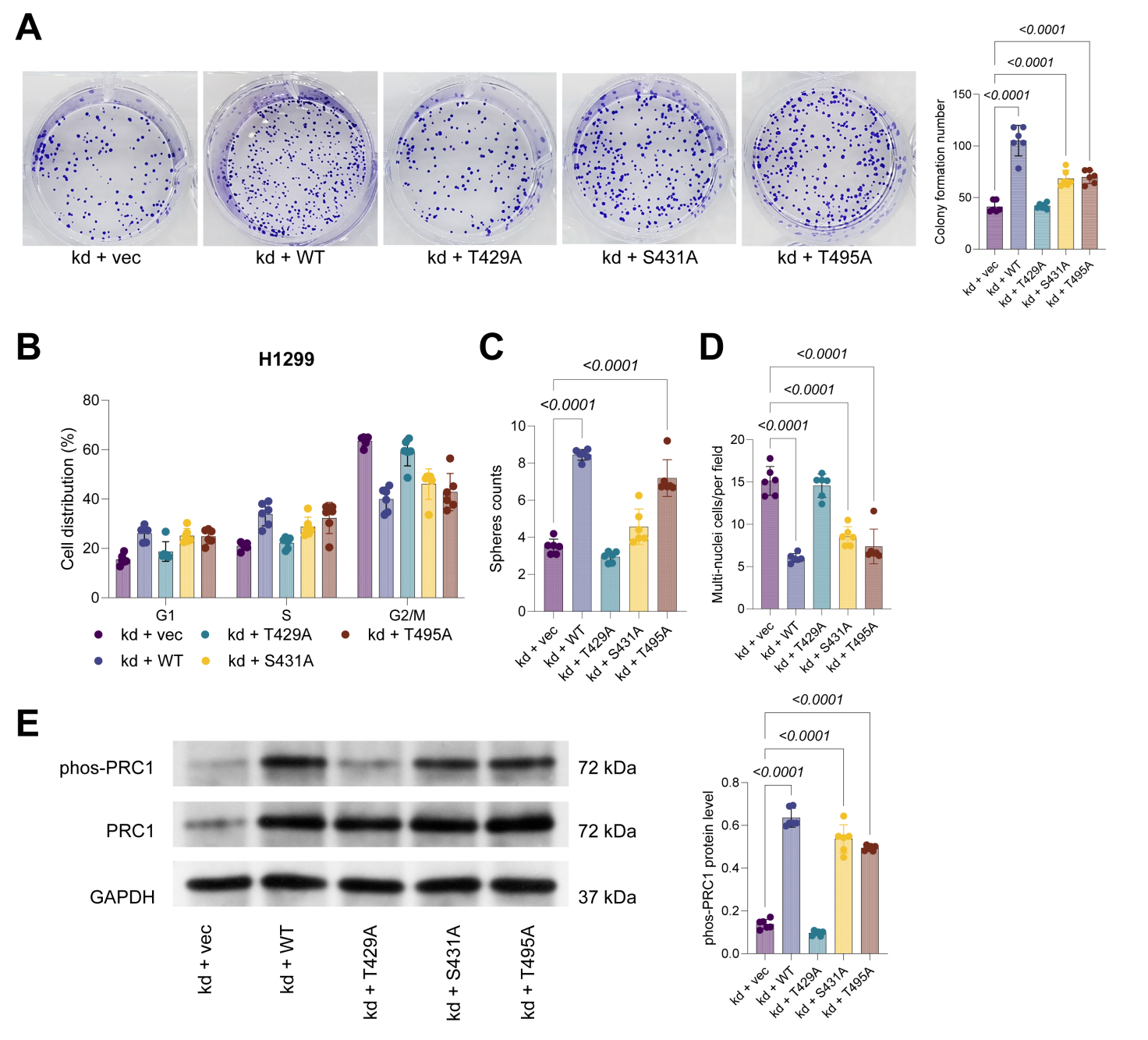


**Fig. S4.** PRC1 phosphorylation at T429 site is significant for its activation and oncogenic roles in NSCLC cells. Following bioinformatic predictions of PRC1 phosphorylation sites, PRC1 (WT, T429A, S431A, and T495A) variants were constructed and loaded into PRC1-kd H1299 cells. A, colony formation ability of cells analyzed using colony formation assay; B, cell cycle distribution in cells analyzed by flow cytometry; C, sphere formation ability of cells analyzed using sphere formation assay; D, number of multinucleated cells analyzed by DAPI staining; E, phosphorylation of PRC1 in cells determined using WB analysis. Cellular experiments were repeated 6 times. Data are presented as dots and bars. The significance level was set at *P* < 0.05.

**
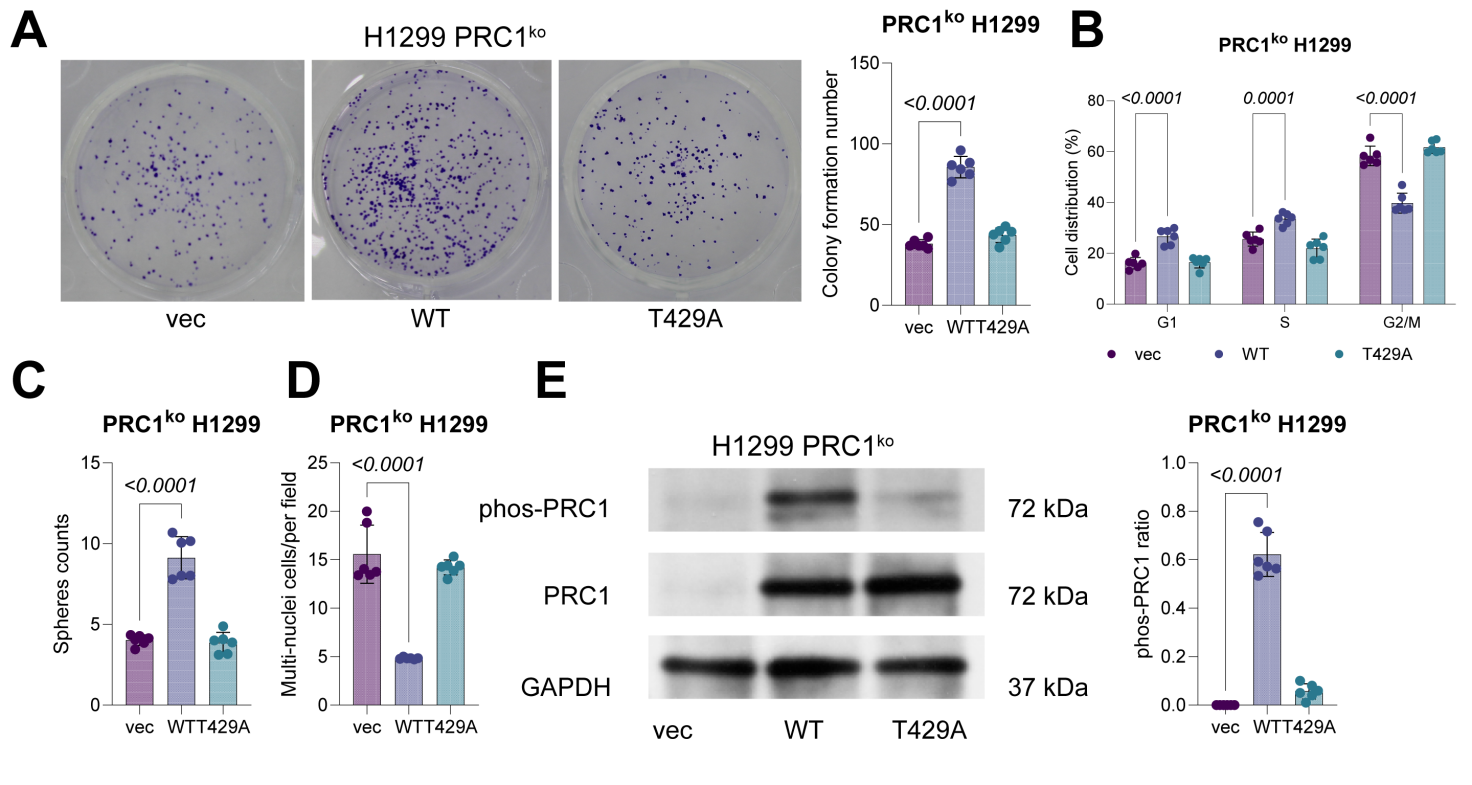
**

**Fig S5** The T429A variant dose not rescue growth activity of PRC^ko^ H1299 cells. PRC1 WT or T429A variant was administered to H1299 PRC1^ko^ cells. A, colony formation ability of cells analyzed using colony formation assay; B, cell cycle distribution in cells analyzed by flow cytometry; C, sphere formation ability of cells analyzed using sphere formation assay; D, number of multinucleated cells analyzed by DAPI staining; E, phosphorylation of PRC1 in cells determined using WB analysis. Cellular experiments were repeated 6 times. Data are presented as dots and bars. The significance level was set at *P* < 0.05.


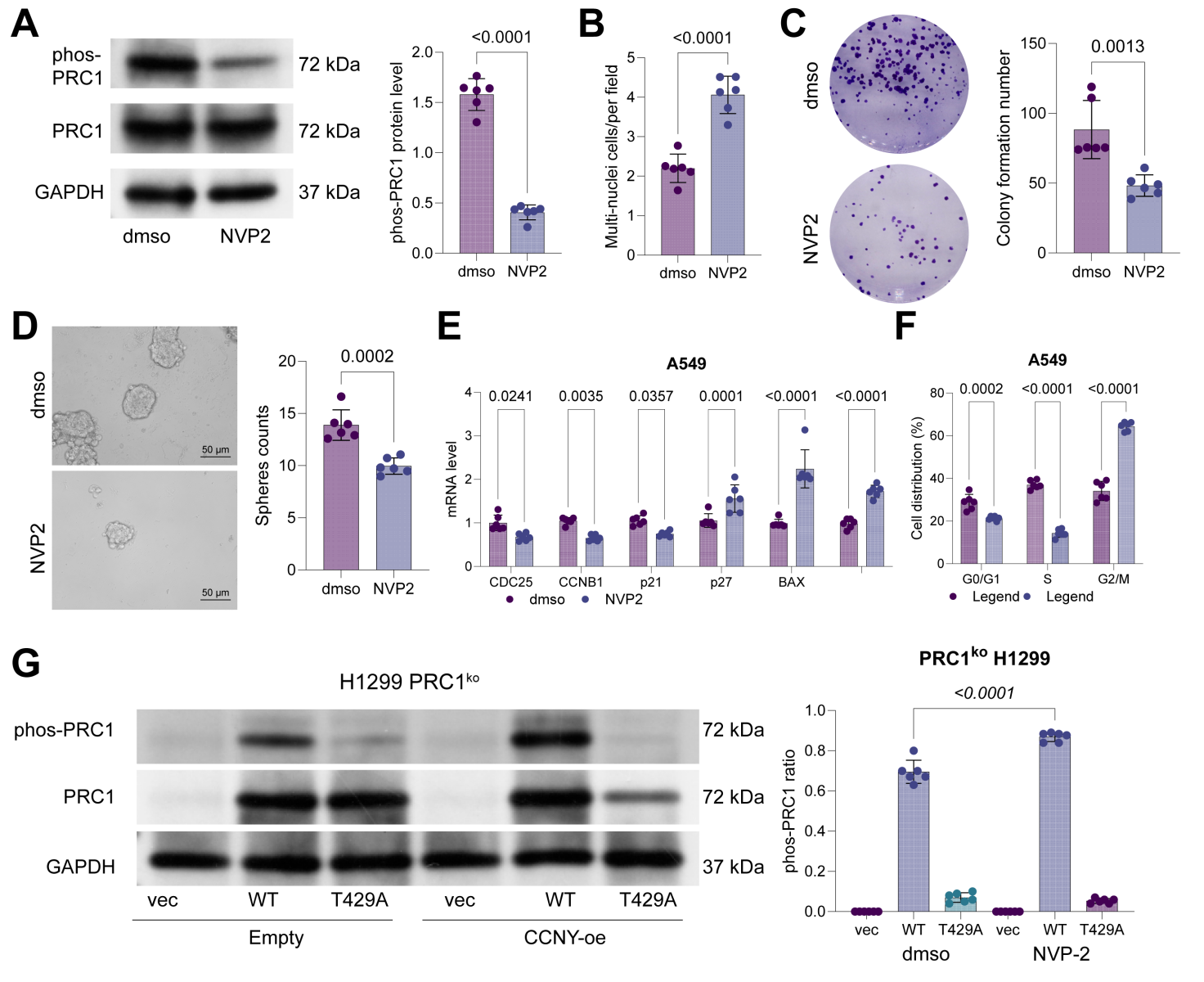


**Fig. S6**. **NVP-2 alleviates malignant phenotypes in A549 cells promoted upon CCNY overexpression.** A549 cells transfected with CCNY-oe were administered with NVP-2, an antagonist of the CDK16/CCNY complex. A, phosphorylation and protein levels of PRC1 in cells determined using WB analysis; B, number of multinucleated cells analyzed by DAPI staining; C, colony formation ability of cells analyzed using colony formation assay; D, sphere formation ability of cells analyzed using sphere formation assay E, mRNA expression of key cell cycle regulators (CDC25A, CCNB1, and CDC2) and apoptosis-related genes (p27, p21, and BAX) determined using qPCR analysis; F, cell cycle distribution in cells analyzed by flow cytometry using sphere formation assay. G, PRC^ko^ H1299 cells administered PRC1 WT or T429A variant were additionally transfected with CCNY-oe plasmid or empty control. G, PRC1 phosphorylation in cells determined using WB analysis. Cellular experiments were repeated 6 times. Data are presented as dots and bars. The significance level was set at *P* < 0.05.


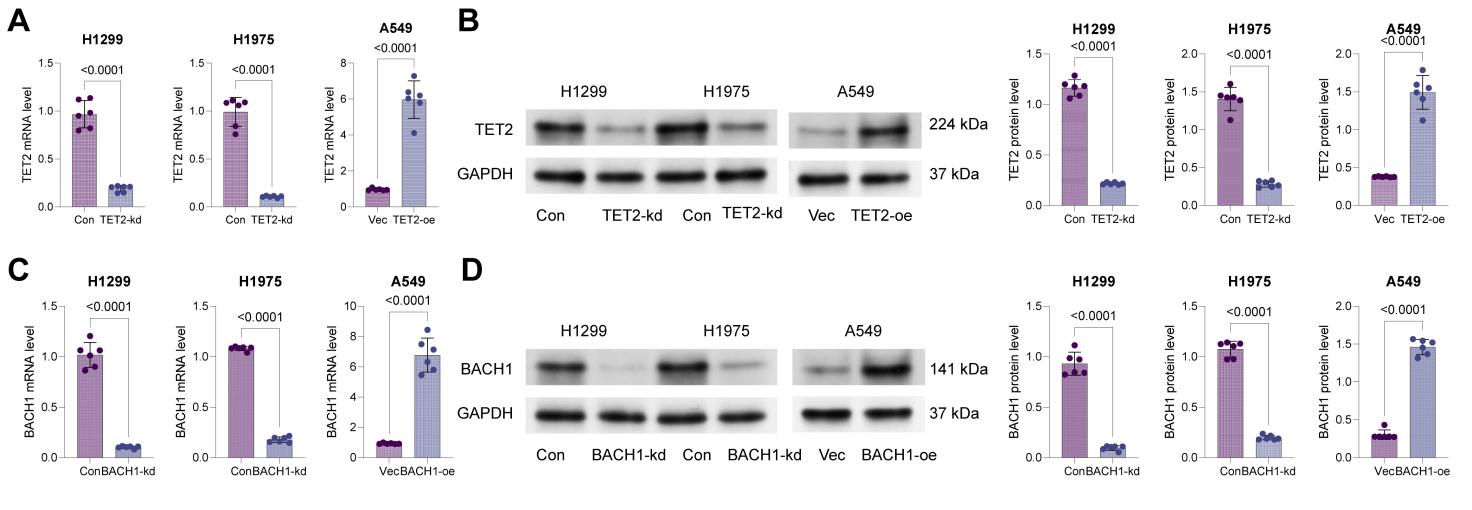


**Fig. S7. Effective gene interference of TET2 and BACH1 in NSCLC cells.** A-B, mRNA (A) and protein (B) levels of TET2 in H1299 and H1975 cells transfected with TET2-kd, or in A549 cells transfected with TET2-oe, or the respective control plasmids, determined using qPCR and WB analyses; C-D, mRNA (C) and protein (D) levels of BACH1 in H1299 and H1975 cells transfected with BACH1-kd, or in A549 cells transfected with BACH1-oe, or the respective control plasmids, determined using qPCR and WB analyses. Cellular experiments were repeated 6 times. Data are presented as dots and bars. The significance level was set at *P* < 0.05.
